# Bioinformatics analysis of ERCC family in pan-cancer and ERCC2 in bladder cancer

**DOI:** 10.3389/fimmu.2024.1402548

**Published:** 2024-08-13

**Authors:** Siyang Zhang, Zhenghui Guan, Qiangqiang Xia, Chong Shen, Hailong Hu, Jiangping Wang

**Affiliations:** ^1^ Department of Urology, The affiliated Taizhou People’s Hospital of Nanjing Medical University, Taizhou School of Clinical Medicine, Nanjing Medical University, Taizhou, Jiangsu, China; ^2^ Postgraduate Training Base of Dalian Medical University, The affiliated Taizhou People’s Hospital of Nanjing Medical University, Taizhou School of Clinical Medicine, Nanjing Medical University, Taizhou, Jiangsu, China; ^3^ Emergency Surgery, Yongcheng People’s Hospital of Henan Province, Henan, Shangqiu, China; ^4^ Department of Urology, Tianjin Institute of Urology, The Second Hospital of Tianjin Medical University, Tianjin, China; ^5^ Tianjin Key Laboratory of Urology, Tianjin Institute of Urology, The Second Hospital of Tianjin Medical University, Tianjin, China

**Keywords:** ERCC, pan-cancer, prognosis, immune infiltration, tumor microenvironment, multi-omics

## Abstract

**Background:**

Single nucleotide polymorphisms (SNPs) in DNA repair genes can impair protein function and hinder DNA repair, leading to genetic instability and increased cancer risk. The Excision Repair Cross-Complementation (ERCC) family plays a crucial role in nucleotide excision repair, yet their comprehensive multi-omics characterization and roles in tumor prognosis and immune microenvironment remain unexplored.

**Methods and materials:**

We performed bioinformatics analysis using publicly available data from 33 cancer types to investigate associations between ERCC gene expression, patient prognosis, and clinical features. We also validated the role of ERCC2 in bladder cancer through *in vitro* assays, including CCK-8, colony formation, wound healing, and Transwell assays.

**Results:**

By utilizing the most recent database, we have conducted an analysis that reveals associations between variations in ERCC expression across multiple cancer types and both patient prognosis and the tumor microenvironment. To ensure the reliability of our findings, we applied the Benjamini-Hochberg procedure to adjust for multiple testing. After correction, we identified that ERCC expression levels remained significantly correlated with patient prognosis in various cancer types (p < 0.05). In addition, according to the results of drug sensitivity studies of anticancer drugs, there is a large correlation between ERCC expression and the sensitivity of different anticancer drugs. Finally, *in vitro* cell behavioral assays determined that knockdown of ERCC2 gene expression significantly inhibited the proliferation, migration and invasion of bladder cancer cells.

**Conclusion:**

Through in-depth exploration of ERCC differential expression and its correlation with immune-related indicators, the unique microenvironment of tumors, and patient prognosis, we verified the potential role of ERCC2 in the process of bladder cancer genesis and progression. Therefore, we believe that the ERCC family of genes is expected to be a new option for cancer treatment and deserves to be further explored in the future.

## 1 Introduction

Cancer remains a leading cause of mortality globally, posing a significant barrier to increasing life expectancy (1). The International Association of Research on Cancer (IARC) reports that cancer is one of the primary causes of death in 112 out of 183 countries (2). In 2020, there were approximately 19.3 million new cancer cases and 10 million deaths, with projections suggesting over 28.4 million cases by 2040 (3). To enhance our understanding of the origins, progression, and management of cancer, it is imperative to identify pivotal genes associated with tumor formation (4). The Cancer Genome Atlas (TCGA) project has significantly advanced our understanding of cancer by providing comprehensive molecular profiles for over 11,000 tumors across 33 different cancer types (5). The identification of crucial genes involved in the process of carcinogenesis can be facilitated through the utilization of extensive sample sizes, high-throughput techniques, and diverse datasets encompassing various types of cancer. Currently, with the development of major medical databases, this has made it possible to conduct comprehensive and in-depth pan-cancer studies on a variety of platforms (6).

DNA damage is significantly correlated with a wide range of cancer-causing agents, including those of environmental and genetic origin. Failure to address or appropriately respond to insults could result in genetic modifications and subsequent development of different types of cancer (7). To minimize harmful damage, DNA repair systems can be used. Genetic polymorphisms, as opposed to mutations, are a substantial type of genetic variation that is more common in persons who have a predisposition to cancer susceptibility. Observations have been made on the functional correlation between genetic variations in different genes related to DNA repair pathways, including the Nucleotide Excision Repair (NER) pathway, and the activity of proteins. These polymorphisms have the potential to decrease the capability of DNA repair, which has been associated with chromosomal instability and the development of cancer (8). It is widely postulated that NER pathway is responsible for the regulation of oxidative DNA damage, repair of adducts, cross-links, and thymidine dimers (9). This repair process is facilitated by the excision repair cross-complementation (ERCC) gene family, with particular emphasis on the involvement of ERCC1 and ERCC2 (10, 11). The association between SNPs and the deficiency of DNA repair capacity in NER genes has been reported. This association may lead to changes in mRNA expression or protein activity (12). The presence of SNPs within DNA repair genes has the potential to hinder protein function and diminish the ability to repair DNA. This can lead to genomic instability and increase an individual’s susceptibility to developing malignancies (13). The gene known as excision repair cross-complementation (ERCC) is responsible for encoding a protein that has the potential to serve as a rate-limiting factor in the nucleotide excision repair pathway (14).

In summary, cancer is a leading cause of mortality worldwide, with DNA repair mechanisms playing a crucial role in preventing carcinogenesis by maintaining genomic stability. Among these mechanisms, the nucleotide excision repair (NER) pathway is vital for repairing bulky DNA lesions. The ERCC (Excision Repair Cross-Complementation) family of genes, including ERCC1-6 and ERCC8, are key components of the NER pathway. Mutations and altered expression of ERCC genes have been implicated in various cancers, suggesting their potential as biomarkers for cancer prognosis and therapy response. Despite the importance of ERCC genes, comprehensive pan-cancer studies of ERCC gene expression patterns and clinical relevance are lacking. Our study aims to fill this gap in terms of differences in ERCC gene expression in different cancer types, the prognostic significance of ERCC gene expression for cancer patients, and how ERCC genes interact with the tumor microenvironment and influence treatment outcomes. To address these questions, we conducted a multi-omics analysis using data from public databases and performed functional assays to validate the role of ERCC2 in bladder cancer. Our study provides novel insights into the role of ERCC genes in cancer progression and highlights their potential as targets for personalized cancer therapy.

## 2 Materials and methods

### 2.1 Data gathering

We utilized data from The Cancer Genome Atlas (TCGA) project, which provides comprehensive molecular profiles for over 11,000 tumors across 33 different cancer types. Patient samples were selected based on the availability of both clinical and genomic data (5). Inclusion criteria included having complete information on ERCC gene expression, clinical outcomes, and relevant clinicopathological features. Samples with missing data or poor-quality genomic profiles were excluded to ensure the reliability of the analysis. These findings have the potential to provide valuable insights for precision oncology (15). Furthermore, a multitude of autonomous researchers have utilized The Cancer Genome Atlas (TCGA) as a valuable asset to bolster their own investigations and facilitate the analysis of molecular testing conducted on individual patients within a clinical context (16). Important data such as transcriptional expression and clinical relevance of 33 pan-cancer tumors were obtained through public data from the UCSC Xena database (17).

### 2.2 Expression analysis of the ERCC genes in pan-cancer

Initially, we eliminated any data that was not related to transcription and kept only the transcriptional data collected from 33 tumors that span across different types of cancer. Our objective was to generate a box plot that would facilitate a comparison of the expression levels of seven distinct ERCC genes (ERCC1-6 and ERCC8). Following that, a statistical analysis was conducted on the results of comparing gene expression in normal and tumor cells across 18 different forms of cancer. This study was confined to cases where the sample size for the normal group was greater than five. In the final phase of this research endeavor, the investigation focused on evaluating the extent of differential expression exhibited by the ERCC genes across 18 distinct types of malignancies. Additionally, the “corrplot” R package was utilized to conduct a more in-depth analysis of the correlation among ERCC genes.

### 2.3 Clinical correlation analysis

The categorization was achieved by comparing their expression levels to the median level of expression of the ERCC genes. The differential analysis was also used to find out how the first treatment affected each cancer and how the mean levels of ERCC expression changed during different stages of the diseases. It was thought that there was statistical significance when the significance level was P < 0.05.

### 2.4 Correlation of ERCC expression with gene mutations

The cBioPortal for cancer genomics, accessible at http://cbioportal.org, is an online tool that allows users to explore, visualize, and analyze complex cancer genomics data. The portal translates data from molecular profiles of cancer tissues and cell lines into a series of events such as genes that can be easily interpreted. Personalized and customized data storage enables researchers to conveniently explore genetic variation and establish links to clinical outcomes when the underlying data is accessible (18). The portal offers many important aids to scientific research, most notably its ability to provide visualizations of gene-level data from multiple platforms. The user-friendly web interface of the portal enables researchers and physicians to access complicated cancer genomics profiles without the need for bioinformatics skills, hence facilitating biological discoveries (19). The dataset used in this investigation was collected from the ICGC/TCGA Pan-Cancer Analysis of Whole Genomes Consortium 2020. It comprised 2683 cases and was sourced via cBioPortal.

### 2.5 Correlation between ERCC expression and tumor microenvironment

Our study aimed to examine the association between the expression of ERCC genes and immunological subtypes across various cancer types. The investigation also encompassed an examination of the immunological and stromal scores associated with each tumor sample. Subsequently, correlation analyses were conducted to correlate the associations between ERCC and immune/stromal scores.

### 2.6 Relationship between ERCC expression and TMB/MSI

The Spearman correlation test was utilized to ascertain the relationships between TMB/MSI and ERCC expression in pan-cancer. Furthermore, this test was employed to assess the correlations between the levels of immunological checkpoints expression and ERCC expression. We then created heatmaps with strong visual effects to better demonstrate the results of the analysis.

### 2.7 A reliable partner for tumor immunity-TIMER

With TIMER 2.0, we performed a multidimensional systematic evaluation of different immune cells. The website can be reached at http://timer.comp-genomics.org/. We then analyzed whether there was a relationship between ERCC and different immune cell.

### 2.8 Relationship between ERCC expression and sensitivity to anticancer drugs

We obtained data on drug sensitivity and other relevant data from the CellMiner database. Afterwards, we then systematically analyzed the relationship between ERCC and drug sensitivity. Ultimately, a correlation was established between the manifestation of ERCC and the responsiveness to pharmaceutical agents.

### 2.9 Gene set enrichment analysis

GSEA was performed to determine the biological role and probable signaling mechanism of ERCC genes in the context of bladder cancer. This objective was achieved by utilizing the KEGG and Hallmark gene sets.

### 2.10 Statistical analysis

The statistical significance between different subgroups was assessed using the log-rank test, and the comparison of survival rates between groups with high and low expression levels was conducted by Kaplan-Meier curve analysis. To adjust for multiple testing, we applied the Benjamini-Hochberg procedure. Additionally, we utilized Cox proportional hazards models with multivariate adjustments for potential confounders, including age, gender, and cancer stage, to verify the prognostic significance of ERCC gene expression. All analyses were performed using R software, with an adjusted p-value < 0.05 considered statistically significant.

## 3 Results

### 3.1 ERCC Expression in Pan-Cancer

**Figure 1:**
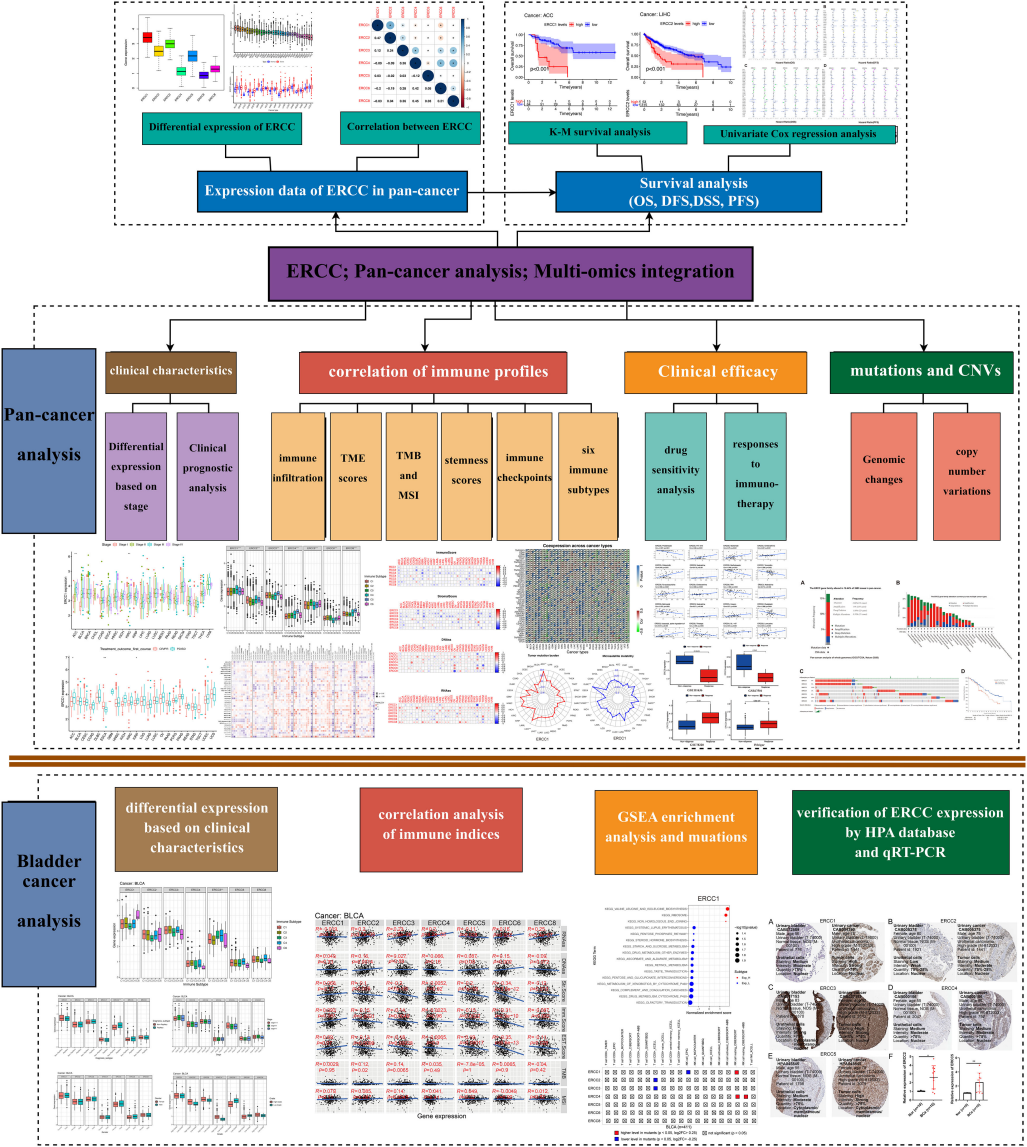
Study workflow. Flowchart illustrating the various stages and processes involved in the study. * means p < 0.05, implying that the result is significant at the 95% confidence level. ** means p < 0.01, meaning that the result is significant at the 99% confidence level. *** means p < 0.001, meaning that the result is significant at the 99.9% confidence level.

**Figure 2:**
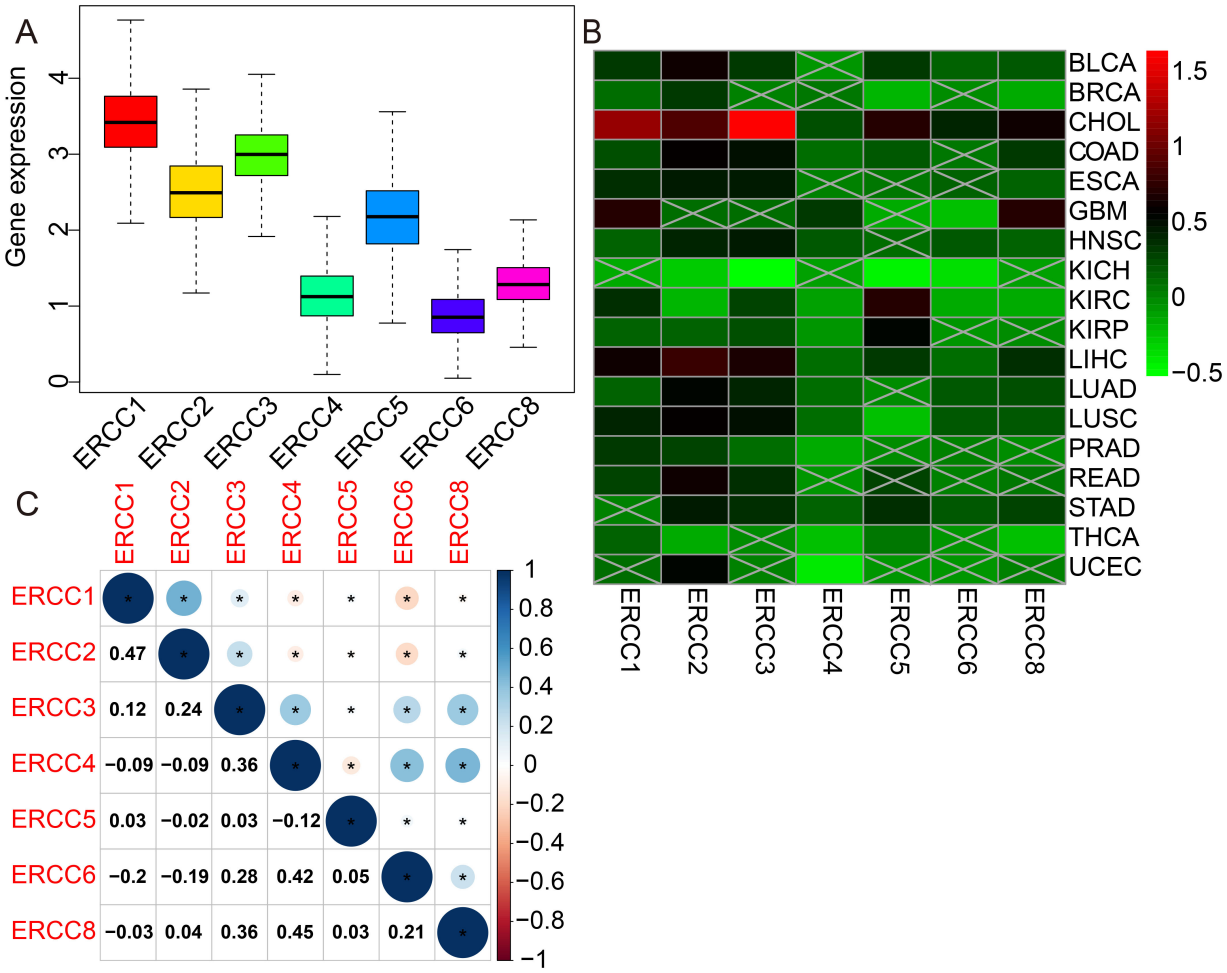
Differential expression of ERCC genes in pan-cancer. **(A)** Box plots showing overall expression levels of ERCC1-6 and ERCC8 across different cancer types. **(B)** Heatmaps illustrating the expression levels of ERCC1-6 and ERCC8 in various cancer types compared to normal tissues. **(C)** Heatmaps depicting the correlation between ERCC gene expressions in different cancer types. * indicates that the data there are statistically significant.

**Figure 1** presents a comprehensive flow chart outlining the various stages and processes involved in this study. Significant variability in ERCC gene expression was observed across different cancer types. Initially, we conducted an examination of mRNA expression levels of seven ERCC genes (ERCC1-6 and ERCC8) in various cancer tissues. This investigation was carried out individually for each gene, and the results were compiled using the TCGA database (**Supplementary Figure S1**). The expression levels of each ERCC gene were integrated across different types of cancers. To validate our findings, we utilized Cox proportional hazards models with multivariate adjustments, accounting for potential confounders such as age, gender, and cancer stage. This approach confirmed the robustness of the observed associations between ERCC expression and clinical outcomes. As depicted in **Figure 2A**, it was observed that ERCC1 exhibited higher expression in cancer compared to the other genes. Conversely, ERCC4, ERCC6, and ERCC8 demonstrated relatively low expression levels. Following that, a significant amount of variation in ERCC genes was detected among 18 different types of tumors. The inquiry subsequently analyzed the contrasting levels of expression of ERCC1-6 and ERCC8 in both cancerous and comparable healthy tissues. A total of 33 cancer samples were included in the analysis, with the exclusion of cancers for which the available normal tissue samples were fewer than 5. According to the data presented in **Figure 2B**, the expression levels of ERCC1 and 3 were found to be significantly elevated in CHOL samples compared to the corresponding normal tissues. The levels of ERCC1–6 and ERCC8 expression in KICH were significantly reduced in comparison to the expression observed in corresponding normal tissues. As illustrated in **Figure 2C**, a notable positive correlation (correlation coefficient = 0.47) is observed between ERCC1 and ERCC2, while a striking negative correlation (correlation coefficient = -0.2) is evident between ERCC1 and ERCC6. The results of this study indicate that there are inherent variations in the expression of ERCC genes, either among different ERCC genes themselves or among various types of cancer. Consequently, it is imperative to conduct comprehensive investigations on each of the ERCC genes, as they have the potential to serve as either oncogenes or anti-oncogenes in various types of cancer. **Supplementary Figure S2** illustrates that several ERCC genes displayed higher levels of expression in distinct forms of cancer. For instance, ERCC1 demonstrated increased expression in cancerous tissues across all examined cancer types, surpassing its expression levels in normal tissues. ERCC2 expression levels were significantly higher in bladder cancer than in its corresponding normal tissues. Certain ERCC genes exhibited relatively low expression levels in specific tumor types. For instance, ERCC4 demonstrated low expression in KIRC, KIRP, PRAD, THCA, and UCEC. The levels of expression of ERCC genes exhibited significant variability within and between different types of cancer. Significantly, there was a lack of consistent pattern of expression seen among the several ERCC genes. For instance, the gene ERCC3 was found to be downregulated only in KICH.

### 3.2 Prognostic significance

**Figure 3:**
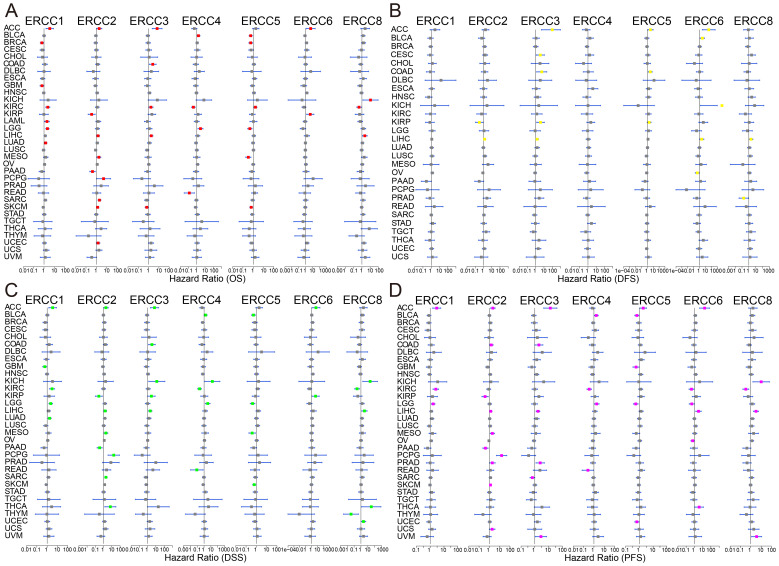
Prognostic significance of ERCC genes. Kaplan-Meier survival curves and univariate Cox regression analysis demonstrating the association between ERCC gene expression and patient survival across various cancers. **(A)** OS, **(B)** DFS, **(C)** DSS, **(D)** PFS.

Univariate Cox regression and Kaplan-Meier survival analyses revealed significant associations between ERCC expression and overall survival (OS), disease-free survival (DFS), disease-specific survival (DSS), and progression-free survival (PFS) across multiple cancer types. As shown in **Figure 3A**, there was a significant correlation between overall survival (OS) time and ERCC expression in multiple cancers. The results of the univariate Cox regression analysis demonstrated comparable prognostic outcomes when applying the same methodology to assess DFS, DSS, and PFS in a cohort of 33 tumors from TCGA dataset (**Figures 3B-D**). The relationship between each gene of ERCC and each cancer type has been further elucidated, as evidenced by the findings presented in **Supplementary Table S1**. For instance, a correlation was observed between decreased expression of ERCC1 and unfavorable overall survival (OS) in ten types of cancers, namely BRCA, CESC, CHOL, ESCA, GBM, HNSC, LUSC, PAAD, PCPG, and PRAD. Conversely, elevated expression of ERCC1 was associated with poor OS in ten different cancers, specifically ACC, KICH, KIRC, LAML, LGG, LIHC, LUAD, MESO, SARC, and UCS. Subsequently, we examined the correlation between ERCC expressions and different stages of cancer. As shown in **Supplementary Figure S3**, a clear negative correlation was observed between the mRNA expression of ERCC4 and the stage of KIRC. This indicates that as the stage of KIRC decreases, there is an increase in the mRNA expression level of ERCC4. According to **Supplementary Figure S4**, following the initial treatment, the levels of ERCC 1, 2, 3, and 6 expressions were observed to be elevated in patients with adrenocortical carcinoma (ACC) who experienced progressive disease/stable disease (PD/SD) compared to those who achieved complete response/partial response (CR/PR). Furthermore, it was observed that patients with LGG exhibited elevated levels of ERCC4 expression in cases of progressive disease or stable disease, compared to cases of complete response or partial response. Conversely, the expression of ERCC5 was found to be lower in cases of progressive disease or stable disease, as opposed to cases of complete response or partial response.

### 3.3 Genomic alterations

**Figure 4:**
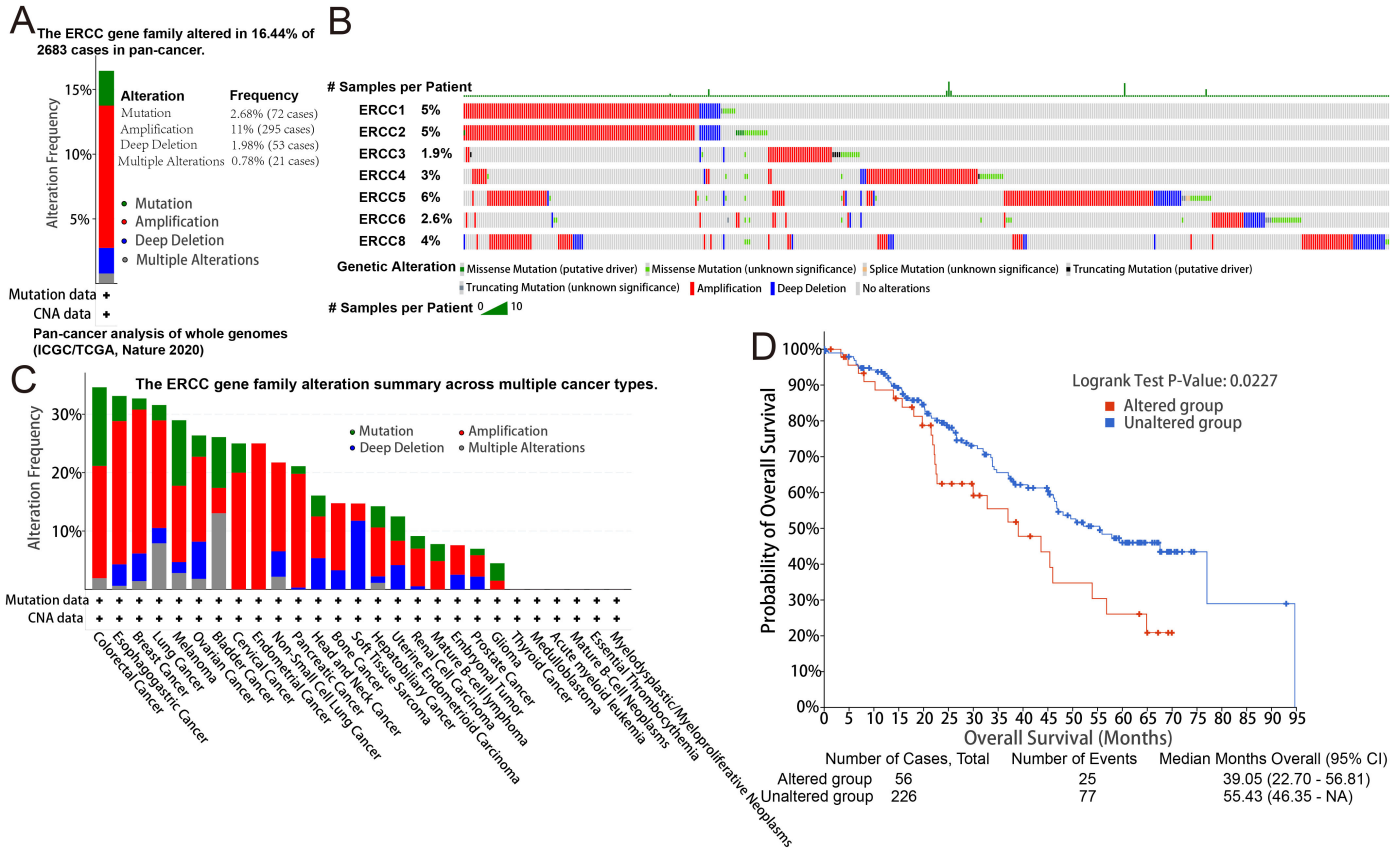
Genetic alterations in ERCC genes. **(A)** Frequency of genetic alterations in ERCC genes across different cancers. **(B)** Types and extent of ERCC gene mutations. **(C)** Summary of mutation types observed in ERCC genes. **(D)** Kaplan-Meier survival curves comparing overall survival between patients with and without ERCC gene alterations.

**Figure 5:**
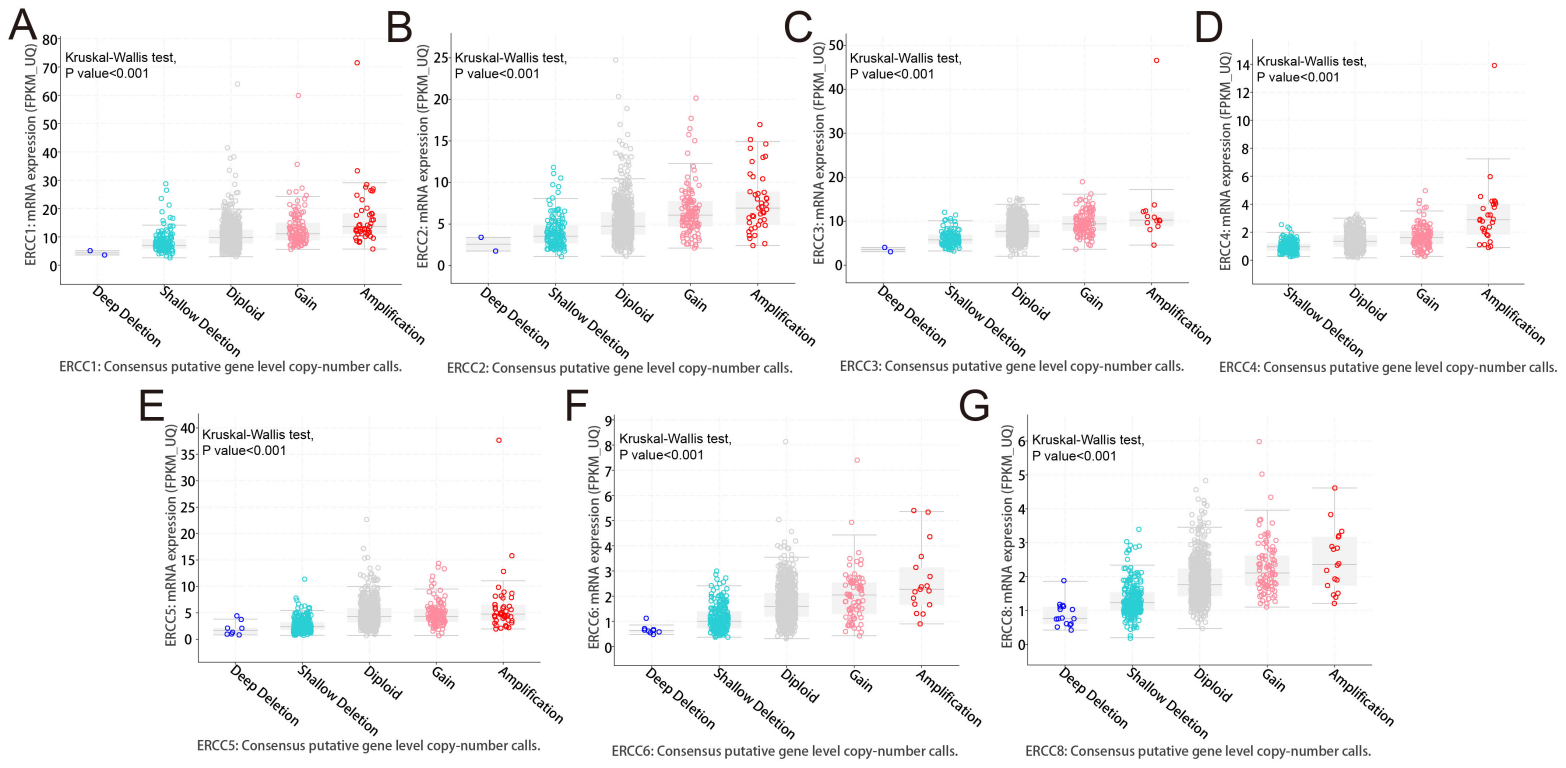
Copy number variations (CNVs) in ERCC genes. Analysis of CNVs in ERCC genes and their correlation with gene expression levels in various cancers. **(A–F)** ERCC1-6, **(G)** ERCC8.

Analysis of targeted sequencing data revealed a high frequency of genetic changes in ERCC genes, with amplifications being the predominant form. Based on the analysis of targeted sequencing data from a cohort of 2683 cancer patients, it was observed that the ERCC gene family exhibited alterations in 16.44% of the cases. Specifically, the ERCC1 gene showed alterations in 5% of the cases, ERCC2 in 5%, ERCC3 in 1.9%, ERCC4 in 3%, ERCC5 in 6%, ERCC6 in 2.6%, and ERCC8 in 4%. Additionally, mutations were detected in 2.68% of the cases (72 cases), amplifications in 11% (295 cases), deep deletions in 1.98% (53 cases), and multiple alterations in 0.78% (21 cases). These findings indicate a high prevalence of mutations within the ERCC gene family (**Figures 4A, B**). As depicted in **Figure 4C**, amplification emerges as the predominant form of genomic alteration within the ERCC gene family, exhibiting detectability across a diverse range of cancer types including lung cancer, endometrial cancer, ovarian cancer, bladder cancer, among others. Patients with alterations in ERCCs in pan-cancer cases may experience reduced therapeutic benefits when stratified based on these alterations. Furthermore, their median overall survival (OS) was significantly lower compared to patients without alterations in ERCC family genes (median OS: 39.05 months versus 55.43 months, P = 0.0227) (**Figure 4D**). **Supplementary Figure S5** contains additional information regarding the mutations of each member of the ERCC family. Based on the findings presented in **Figure 5**, it can be observed that patients with pan-cancer exhibit a higher prevalence of amplification and gain. Additionally, it is evident that the majority of ERCCs expressions show an upward trend as the copy number of CNV increases. Additionally, it was noted that the proportion of non-mutated regions was greater than that of missense mutations (**Supplementary Figure S6**). The findings of the analyses suggest that copy number variations (CNVs) in pan-cancer are predominantly associated with the upregulation of ERCC genes.

### 3.4 Tumor microenvironment

**Figure 6:**
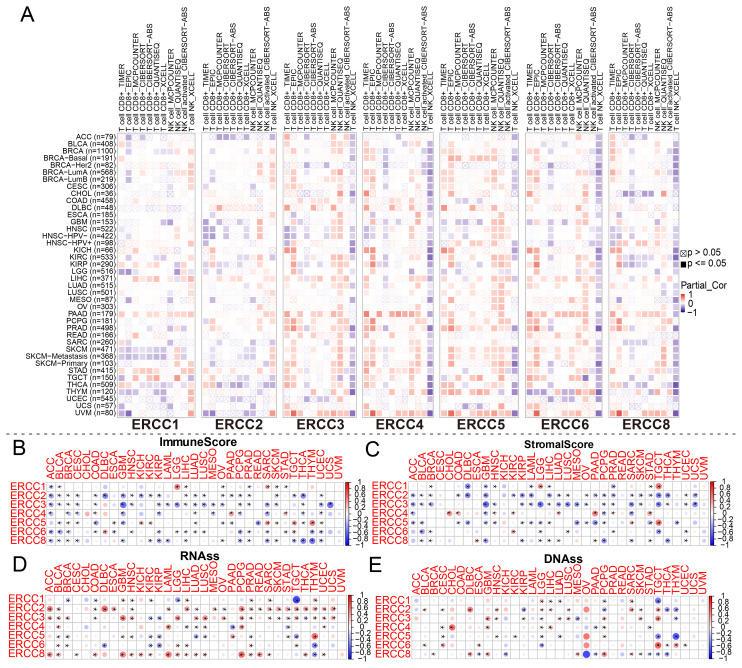
Association between ERCC and immune-related indicators. **(A)** Correlation between ERCC gene expression and immune cell infiltration. **(B–E)** Relationships between ERCC gene expression and ImmuneScore, StromalScore, RNAss, and DNAss, respectively. * indicates that the data there are statistically significant.

As shown in **Figure 6A**, we used spearman correlation heatmaps to investigate the correlation between immune cell infiltration and ERCC expression in tumors. Research on the tumor immune microenvironment has garnered significant attention in recent years due to its potential to enhance our understanding of carcinogenesis, development, diagnosis, prevention, and predictive survival in patients. Upon analyzing the immune-related scores of each tumor sample, we discovered a notable relationship between ERCC differential expression and immune-related scores. To provide precise details, it was observed that ERCC2 exhibited a negative correlation with a majority of the 33 types of cancers (**Figures 6B, C**). This implies that an elevated expression of ERCC2 was associated with a reduced presence of immune cells within the tumors of said patients. Finally, in this study, RNA sequencing (RNAss) and DNA sequencing (DNAss) techniques were employed to investigate the correlation between the ERCC gene family and tumor stemness indices across various types of cancer. Various cancer types have been associated with the involvement of different members of the ERCC family in RNA and DNA processing. As shown in **Figure 6D**, there was a positive correlation between the expression of ERCC2 and most carcinomas, with only a mild negative correlation observed with OV. Similarly, **Figure 6E** demonstrated that at the DNA stem cell index level, the expression of ERCC2, 5, and 6 exhibited a significant positive correlation with OV, while showing a significant negative correlation with ERCC8.

### 3.5 Relationship between ERCC expression and tumor microenvironment-related indicators

**Figure 7:**
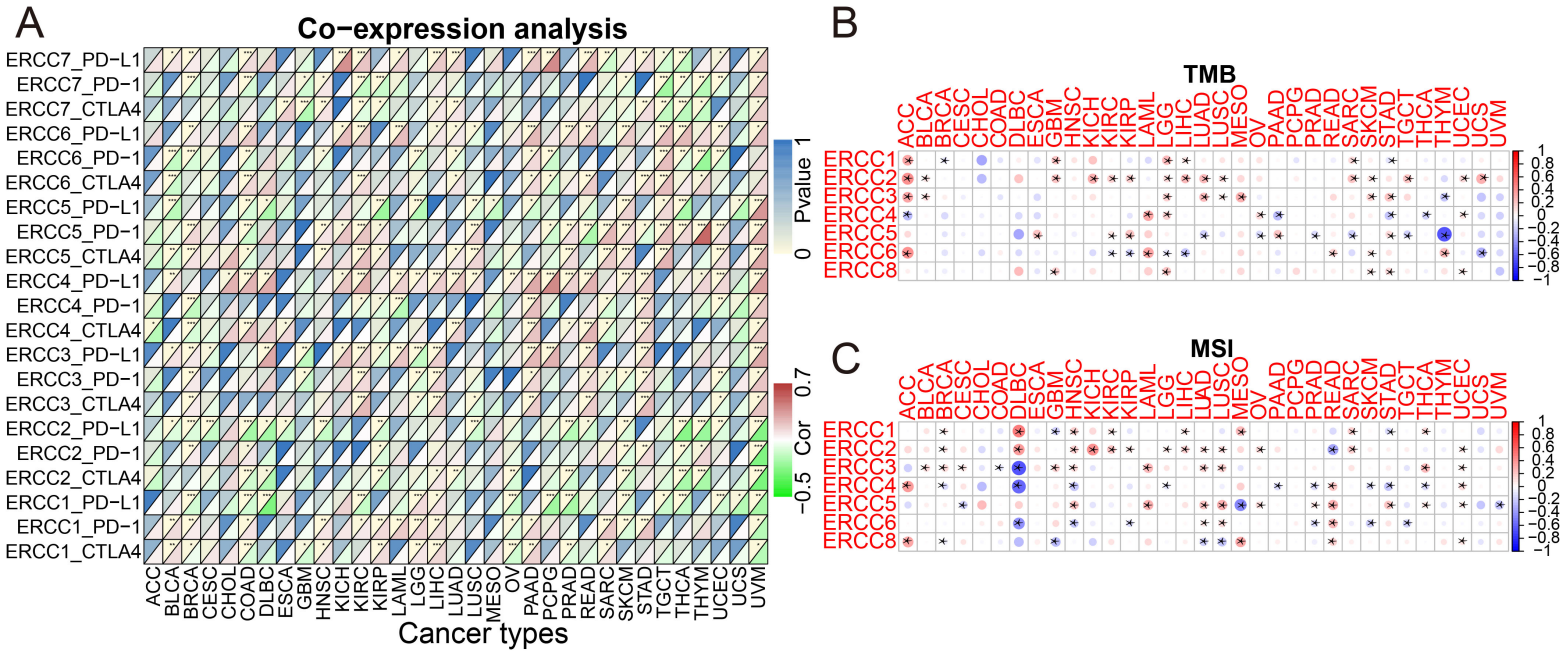
ERCC gene associations with immune prediction scores. **(A)** Correlation between ERCC gene expression and immune checkpoint markers. **(B)** Association of ERCC gene expression with Tumor Mutational Burden (TMB). **(C)** Association of ERCC gene expression with Microsatellite Instability (MSI). * means p < 0.05, implying that the result is significant at the 95% confidence level. ** means p < 0.01, meaning that the result is significant at the 99% confidence level. *** means p < 0.001, meaning that the result is significant at the 99.9% confidence level.

Tumor immunotherapy has gained significant attention in recent years since it is closely associated with the use of immunosurveillance to predict predictive survival in cancer patients (20). We identified a set of three immune checkpoints, in order to investigate the relationship between ERCC expression and immune checkpoints. Our analysis revealed a relationship between ERCC differential expression and immune checkpoints (**Figure 7A**; **Supplementary Figure S7**). Tumor microenvironment (TMB) and microsatellite instability (MSI) are widely recognized as pivotal biomarkers in the context of tumor progression and carcinogenesis. TMB, an acronym for tumor mutational burden, refers to the density of nonsynonymous mutations within the protein coding region. This metric is closely associated with both tumor prognosis and the response to immunotherapy. We found that ERCC1 expression was significantly associated with TMB in ACC, GBM, KICH, STAD, SARC, LIHC, and LGG (**Figure 7B**; **Supplementary Figure S8**). Microsatellite instability (MSI) is a genetic condition characterized by the presence of insertion or deletion mutations that result in variable lengths of the microsatellite (MS) sequence during DNA replication. Functional problems with the mismatch repair system (MMRs) are a common cause of this phenomenon. It was observed that ERCC1 expression exhibited a significant correlation with MSI in various cancer types, including THCA, STAD, SARC, MESO, LIHC, KIRC, HNSC, GBM, DLBC, and BRCA. This finding is illustrated in **Figure 7C** and **Supplementary Figure S9**.

### 3.6 Drug sensitivity analysis

**Figure 8:**
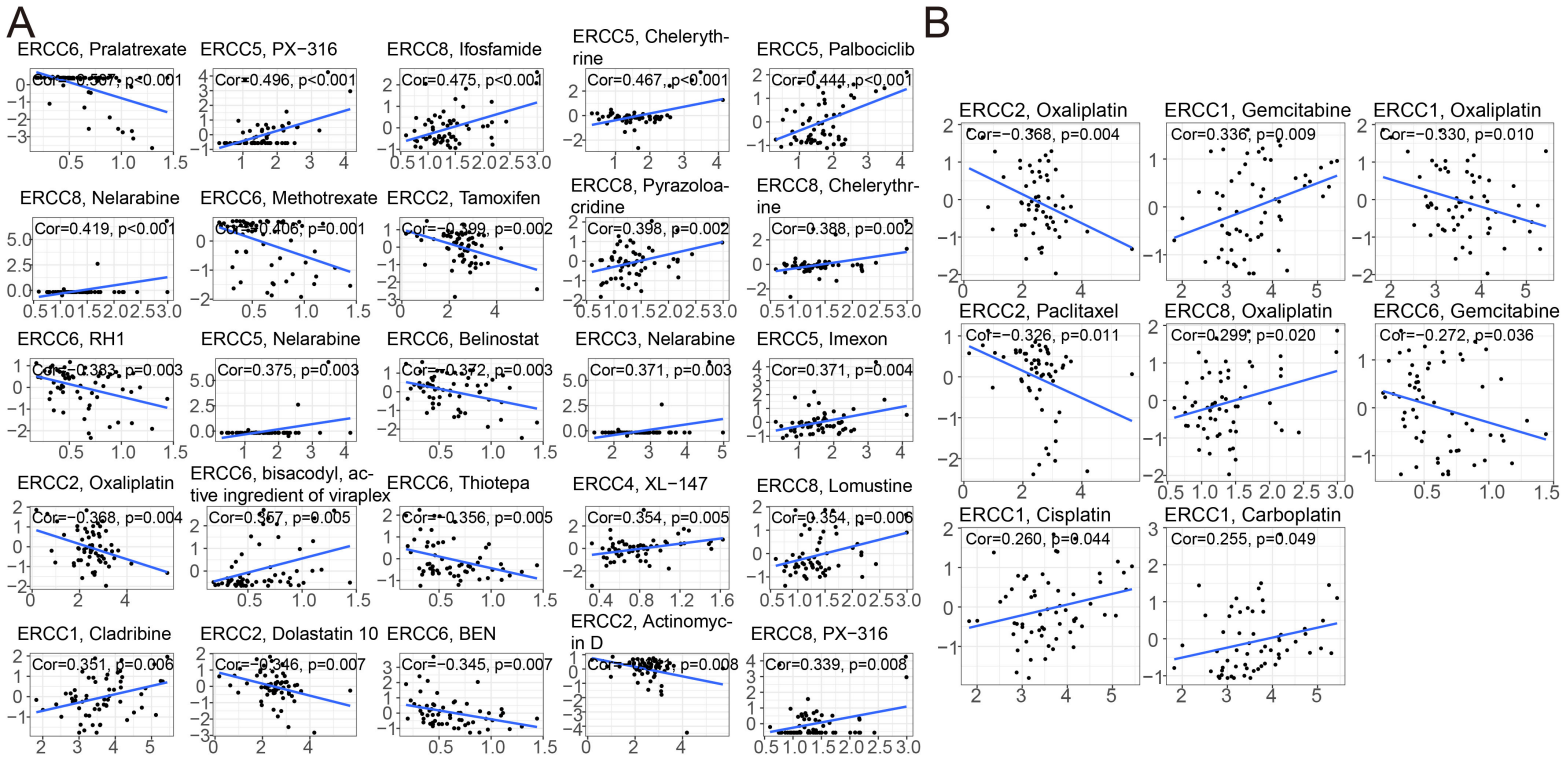
ERCC gene correlation with drug sensitivity. **(A)** Correlation analysis between ERCC gene expression and sensitivity to various anticancer drugs. **(B)** Correlation between ERCC gene expression and responsiveness to different chemotherapeutic agents.

To investigate the relationship between drug sensitivity and ERCC differential expression, we conducted comprehensive studies. The significance of ERCC family genes in cancer therapy resistance has gained increasing attention in the public sphere. The results revealed a significant correlation between ERCC and various medication sensitivities. According to the findings depicted in **Figure 8** (p < 0.05), a positive correlation was observed between ERCC5 expression and the therapeutic effectiveness of PX-316, Chelerythrine, Palbociclib, Nelarabine, and Imexon. This implies that patients exhibiting higher levels of ERCC5 expression demonstrated improved treatment outcomes when administered these medications. Furthermore, a negative correlation was obtained between ERCC6 and the expression of Pralatrexate, Methotrexate, RH1, Belinostat, and Thiotepa. This suggests that patients with elevated ERCC6 expression should exercise caution when considering treatment with these drugs, as higher levels of ERCC6 expression are associated with reduced efficacy of these medications.

### 3.7 Relationship between ERCC expression and clinical immunotherapy efficacy

**Figure 9:**
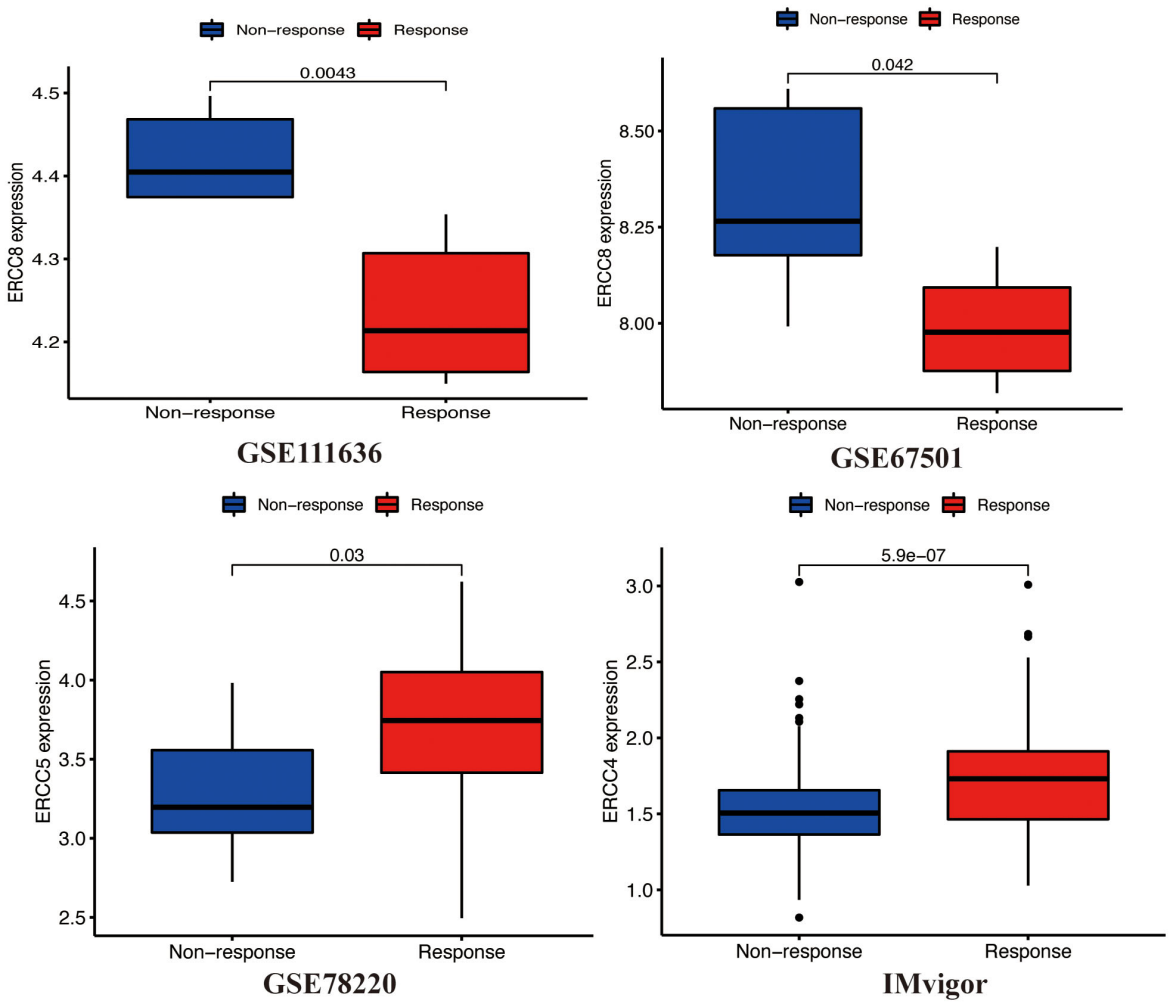
ERCC gene expression post-treatment. Analysis of differential expression of ERCC genes after various cancer treatments.

Several clinical trials have demonstrated that immune checkpoint inhibitors exhibit durable responses and have acceptable safety profiles over the long term. Nevertheless, it is worth noting that a significant proportion, ranging from 70% to 80%, of patients may continue to exhibit a lack of response to immune checkpoint inhibition. Therefore, further research is necessary to investigate the potential synergistic effects that may arise from the integration of immune checkpoint inhibitor therapy with other therapeutic modalities. This exploration aims to enhance the effectiveness of immunotherapies (21). In order to determine the influence of ERCC expression on the success of immunotherapy in different groups, we conducted a study to examine the relationship between ERCC gene expression and the effectiveness of immunotherapy in bladder cancer (GSE111636 and IMvigor), melanoma (GSE78220), and renal cell carcinoma (GSE67501) cohorts. A favorable connection was consistently seen between the expression levels of ERCC4 and ERCC5 and the objective responses to anti-PD-1/PD-L1 treatment in both the Imvigor210 and GSE78220 cohorts (**Figure 9**). In contrast, it was observed that ERCC8 exhibited a positive correlation with resistance to cancer immunotherapy (specifically, anti-PD-1/PD-L1) in the datasets GSE111636 and GSE67501. In summary, the findings of this study suggest that the expression of ERCC genes may serve as a valuable predictor for the response to anti-PD-1/PD-L1 immunotherapy.

### 3.8 GSEA analysis in bladder cancer

GSEA was used to study ERCC-related molecular pathways. Using this as a basis, we identified Hallmark and KEGG pathways that appear enriched in each ERCC. Based on the results obtained from statistical analysis using P value and FDR adjusted p-value, it was observed that a diverse range of tumor-related and immune-related Hallmark and KEGG pathways exhibited differential enrichment across various human cancers, with varying levels of ERCC expression (**Supplementary Figure S10**). The presence of inconsistent results across various tumor types implies that ERCC genes may be involved in tumor progression through diverse mechanisms.

### 3.9 Clinicopathological features in bladder cancer

The work aimed to investigate the relationship between ERCC genes and tumor immunity, specifically examining the potential connection between immune cells and ERCC genes. Previous studies have demonstrated that human cancers exhibit six distinct immunological subtypes (C1-C6), each of which has the potential to either facilitate or impede tumor progression. The stages of wound healing are represented by C1-C6, with each stage corresponding to specific immunological processes. C1 signifies the dominance of interferon-r in the immune response, while C2 represents the inflammatory phase. C3 is characterized by lymphocyte failure, followed by C4 which indicates immune quiescence. In conclusion, the presence of C5 is linked to the prevalence of transforming growth factor-beta (TGF-) during the process of wound healing. Prior studies have demonstrated that individuals exhibiting immunological subtypes C3 and C5 exhibited significantly higher rates of survival in comparison to those with alternative subtypes, namely C4 and C6, which displayed the lowest rates of survival (22). The expression levels of the ERCC gene varied significantly across C1–C6, as depicted in **Supplementary Figure S13A**. The above findings suggest that ERCC is able to recognize several immune subtypes in cancer, thus aiding in cancer diagnosis. In order to obtain a deeper understanding, we examined the correlation between ERCC expressions and the extent of immune cell infiltration in pan-cancer. Further examination could reveal that ERCC1, 2, 3, 4, and 8 expressions were significantly higher in the C4 and 5 subtype compared to the other subtypes, implying a potential association between these genes and the promotion of cancer. In addition, we conducted a thorough examination of the relationship between ERCC and distinct clinicopathological characteristics in BLCA patients. The characteristics encompass tumor grade, and tumor subtype, among other factors (**Supplementary Figures S13B, C**). Our investigation revealed that several factors exert a substantial influence on the ERCC expression.

### 3.10 Tumor microenvironment in bladder cancer

**Figure 10:**
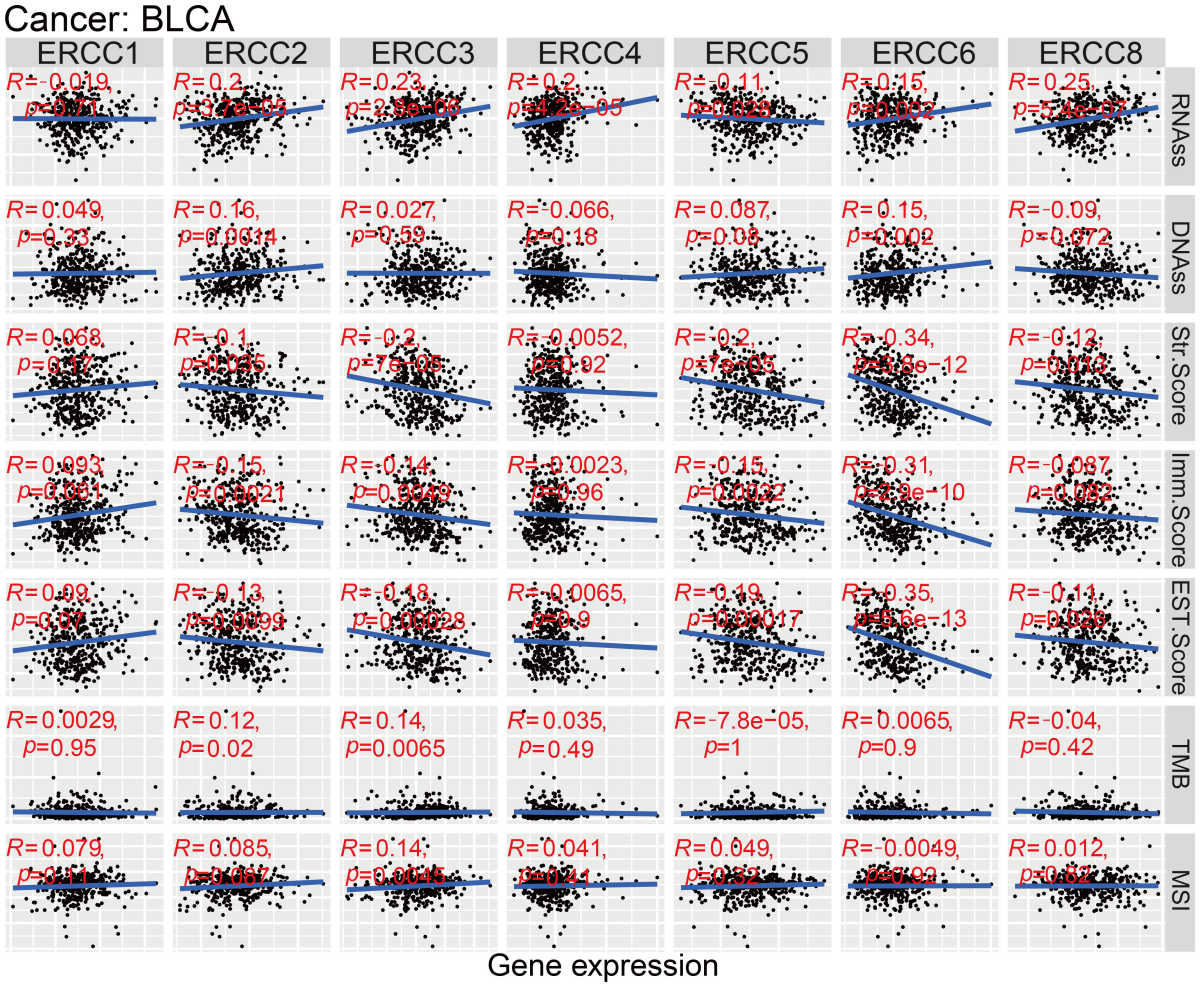
ERCC genes in bladder cancer (BLCA). Multidimensional correlation analysis of ERCC gene expression and various factors in bladder cancer.

**Figure 11:**
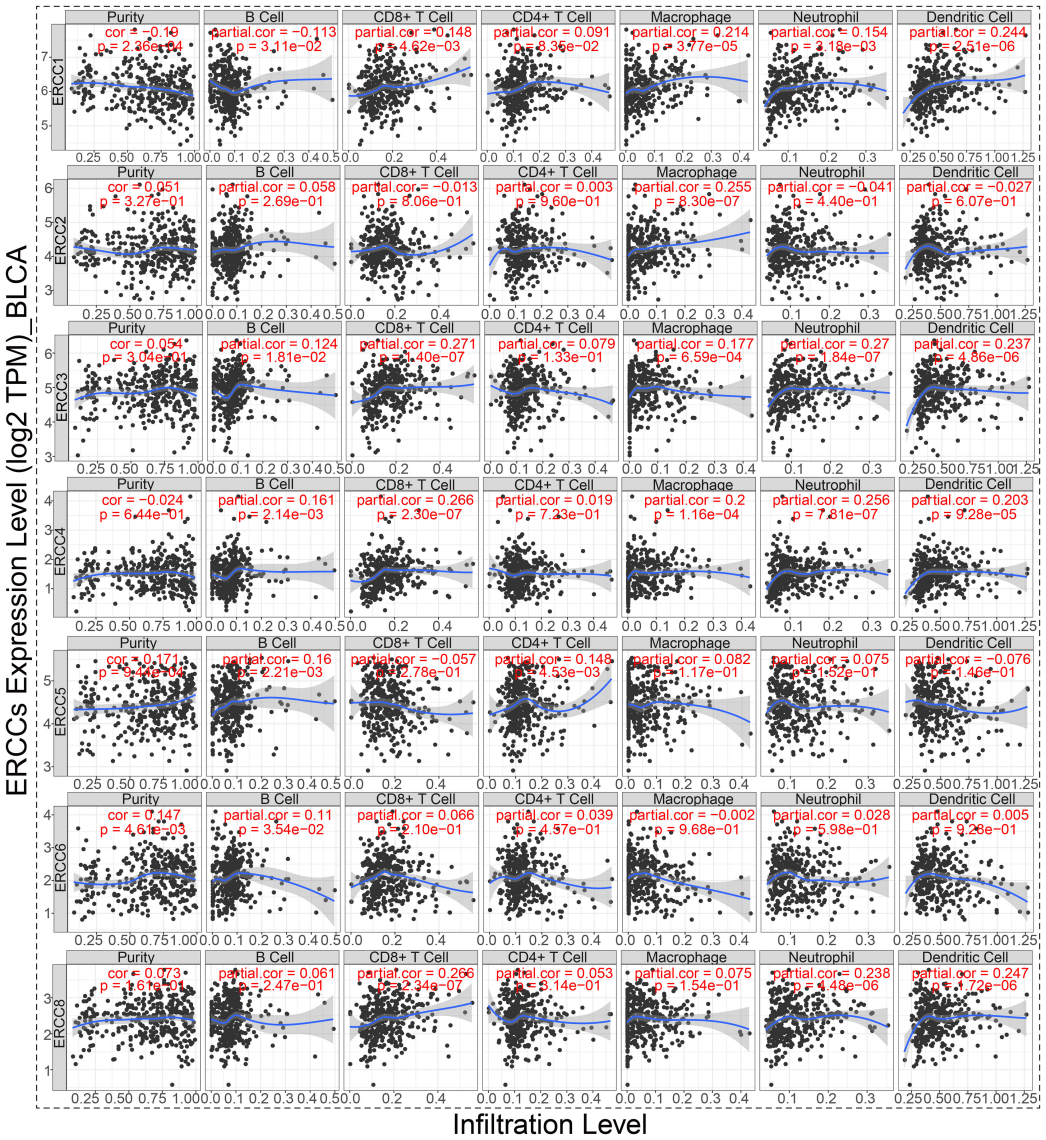
ERCC gene correlation with immune cells in BLCA. Correlation analysis between ERCC gene expression and infiltration of six different types of immune cells in bladder cancer.

As shown in **Figure 10**, there is a significant positive relationship between the expression of ERCC2, 3, 4, 6, and 8 and the BLCA stemness indices in BLCA patients, as evaluated by RNAss. Conversely, ERCC5 exhibits a negative correlation with RNAss (p < 0.05), while the association between ERCC1 and RNAss did not reach statistical significance. In our study, we found that ERCC 2 and 6 were significantly positively correlated with DNAss (p < 0.05). Nevertheless, the analysis did not reveal any statistically significant association between other ERCCs and DNAss (p > 0.05). According to the data presented in **Figure 11**, there is a positive correlation between the expression of ERCC1, 2, 3, and 8 and the infiltration of CD8+T cells. Conversely, ERCC4, 5, and 6 exhibit a negative correlation with the infiltration of CD8+T cells. The findings of our investigation suggest that ERCCs may have the capacity to regulate the infiltration of immune cells in pan-cancer, which could have implications for the overall survival outcomes of individuals.

### 3.11 Correlation analysis of gene mutations in bladder cancer

**Figure 12:**
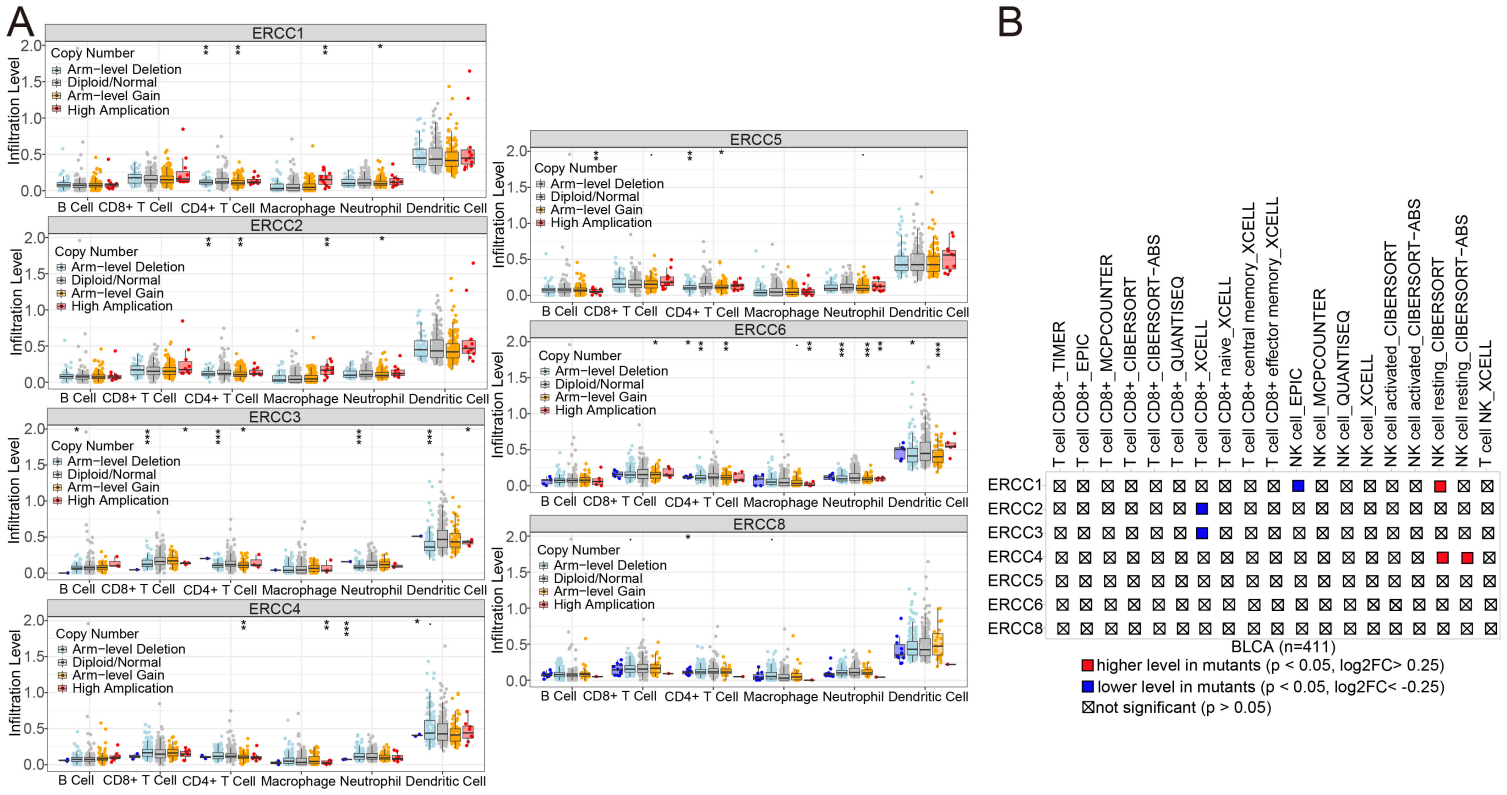
ERCC gene variants and immune infiltration in BLCA. **(A)** Association of ERCC gene copy number variants with immune cell infiltration in bladder cancer. **(B)** Impact of ERCC gene mutations on immune cell infiltration in bladder cancer.

To further understand how ERCC affects immunotherapy, we conducted an investigation into the association between ERCC copy numbers and the abundance of commonly observed immune cells. The presence of ERCC3 and ERCC4 deletions, along with the amplification of ERCC1, ERCC2, ERCC5, and ERCC6 at the arm level, as well as the high amplification of ERCC8, were found to be significantly correlated with a notable decrease in the abundance of CD8+ T cells (refer to **Figure 12A**). As illustrated in **Figure 12B**, the mutant group demonstrates a higher cellular volume in several immune cells in comparison to the non-mutant group. In this instance, it has been observed that the presence of a mutation in the ERCC1 gene leads to an increased quantity of resting NK cells as determined by CIBERSORT analysis. Conversely, non-mutant ERCC1 exhibits a higher level of NK cells as determined by EPIC analysis in comparison to mutant ERCC1.

### 3.12 Verification of ERCC family gene expression by HPA database and qRT-PCR

**Figure 13:**
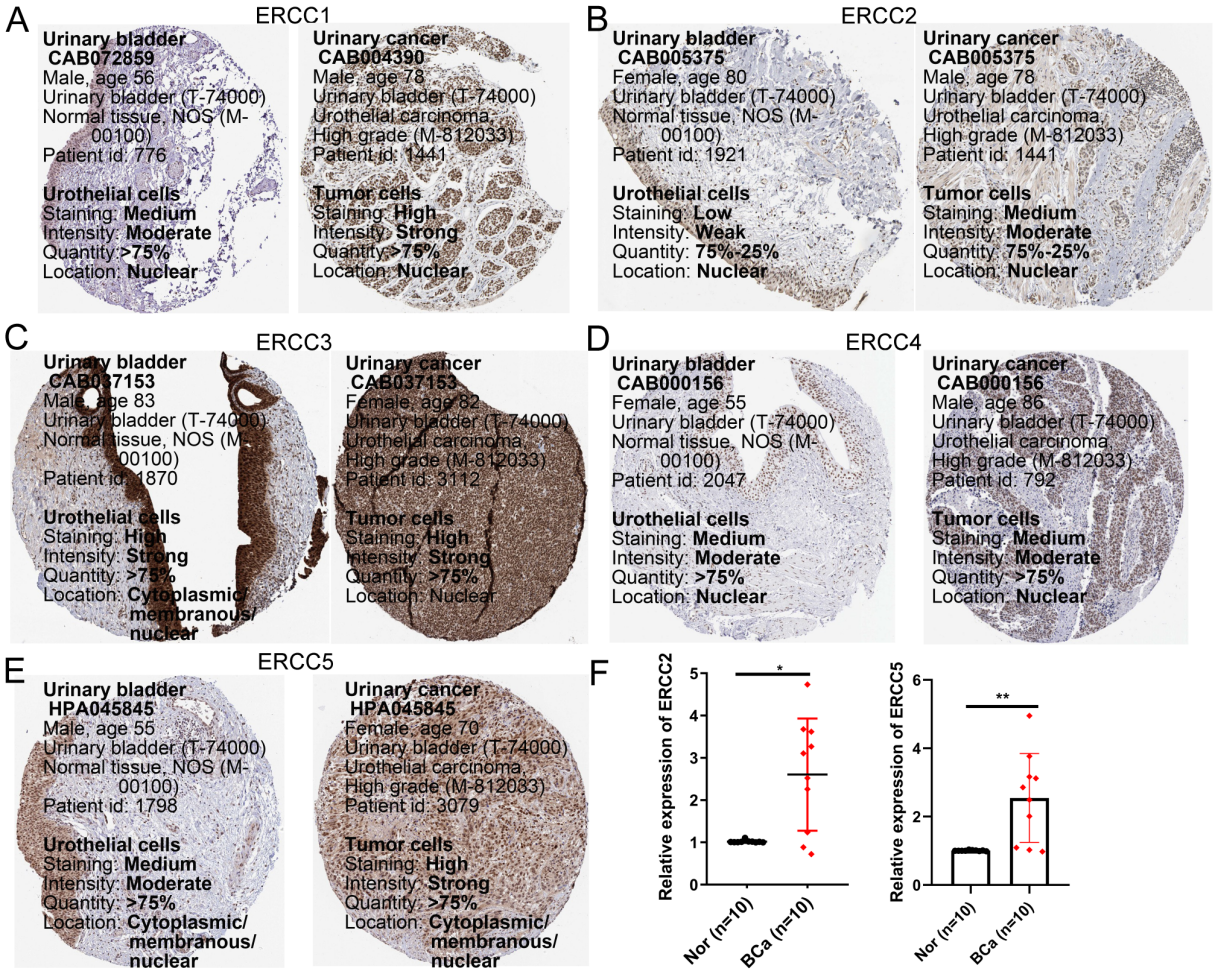
Validation of gene expression of selected members of the ERCC family. **(A–E)** Protein expression levels of ERCC1, ERCC2, ERCC3, ERCC4, and ERCC5 in bladder cancer versus normal tissues from the Human Protein Atlas (HPA) database. **(F)** mRNA expression levels of ERCC2 and ERCC5 in bladder cancer and normal tissues validated by qRT-PCR. * means p < 0.05, implying that the result is significant at the 95% confidence level. ** means p < 0.01, meaning that the result is significant at the 99% confidence level.

We analyzed the protein expression levels of ERCC1, 2, 3, 4, and 5 using human protein atlas (HPA). As shown in **Figures 13A-E**, protein expression of ERCC1, 2, and 5 was up-regulated in bladder cancer tissues and EGR1 was down-regulated in cancer tissues compared to normal bladder samples. Subsequently, mRNA expression levels of ERCC2 and 5 were examined in bladder cancer tissues and normal bladder tissues. As shown in **Figure 13F**, the qRT-PCR results for these genes were similar to the protein expression results described above.

### 3.13 Loss-of-function ERCC2 inhibits the proliferation of bladder cancer cells

**Figure 14:**
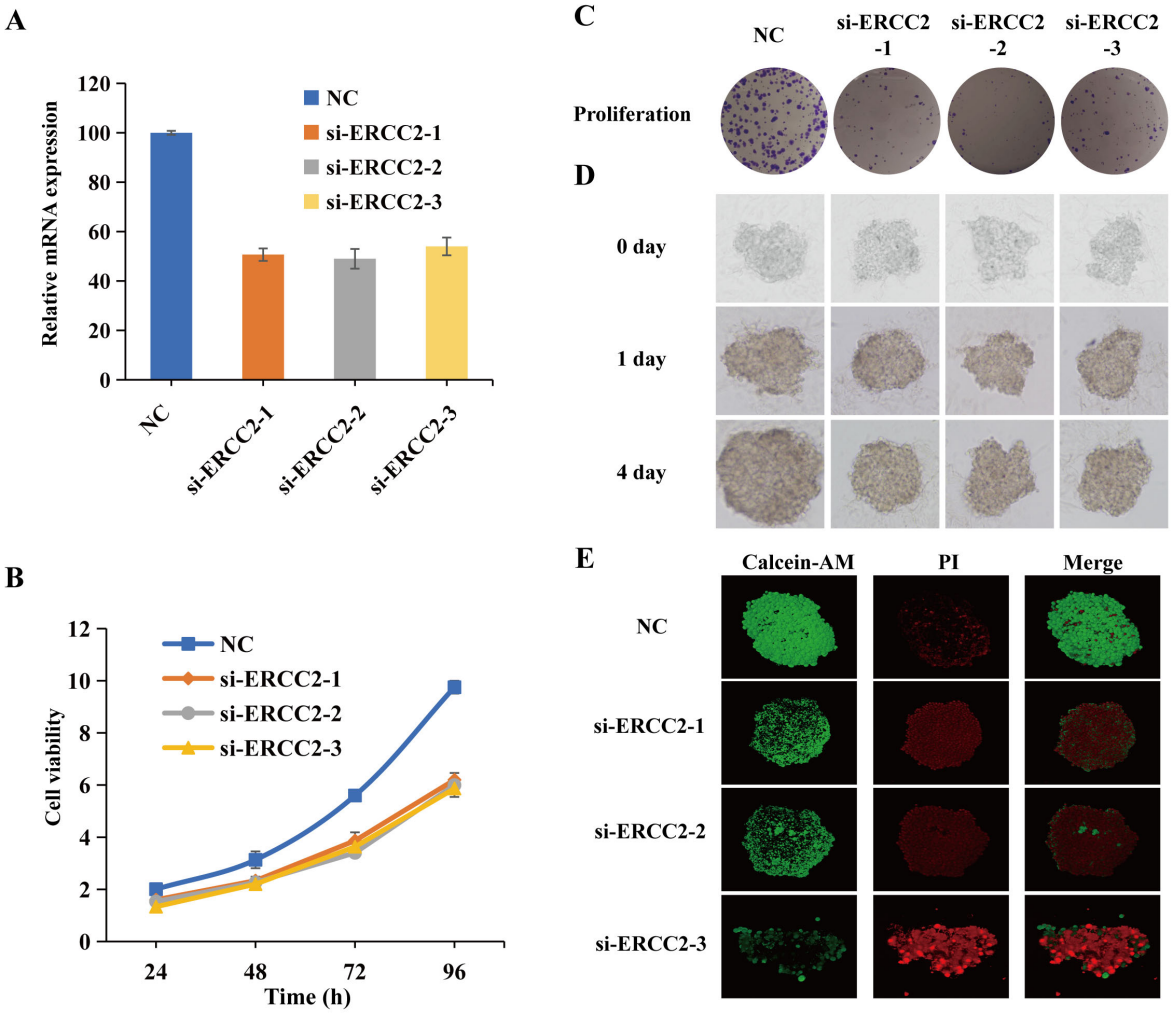
Loss-of-function ERCC2 inhibits the proliferation of bladder cancer cells. **(A)** ERCC2 knockdown efficiency using si-ERCC2-1, si-ERCC2-2, si-ERCC2-3. **(B)** CCK-8 assay shows reduced proliferation in ERCC2 knockdown cells vs. control at 24, 48, 72, and 96 hours. **(C)** Colony formation assay shows fewer colonies in ERCC2 knockdown cells vs. control. **(D)** 3D multicellular spheres show reduced growth in ERCC2 knockdown cells vs. control. **(E)** Live/dead analysis with calcein-AM/PI co-staining confirms inhibited proliferation in ERCC2 knockdown cells.

To investigate the role of ERCC2 in cell proliferation in bladder cancer cells, three siRNAs (si-ERCC2-1, si-ERCC2-2, si-ERCC2-3) were designed to silence ERCC2 expression in T24 cells. As shown in **Figure 14A**, all the above three siRNAs could effectively knockdown ERCC2 expression in T24 cells. To explore the effect of ERCC2 on bladder cancer T24 cells, we performed CCK-8 assay, colony formation assay, and three-dimensional multicellular spheres on the treated T24 cells. The CCK-8 results showed that the proliferative capacity of the experimental group in which ERCC2 was knocked down was significantly lower than that of the T24 cells in the NC group at 24, 48, 72, and 96 hour (p<0.05, **Figure 14B**). As shown in **Figure 14C**, the results of colony formation experiments were similar to those of CCK8 experiments, and the proliferation capacity of cells in the NC group was significantly greater than that in the experimental group. Then three-dimensional (3D) multicellular spheres were introduced to continue to explore the effect of ERCC2 on the proliferation ability of T24 cells. As shown in **Figure 14D**, after prolonged incubation, the 3D multicellular spheres in the control group still maintained rapid growth, whereas the spheres in the experimental group did not show any significant growth, and the proliferative ability was weakened. In addition, live/dead analysis by applying calcein-AM/PI co-staining revealed the effectiveness of ERCC2’s loss of function in inhibiting the proliferation of T24 cells (**Figure 14E**).

### 3.14 Knockdown of ERCC2 inhibits migration and invasion of bladder cancer cells

**Figure 15:**
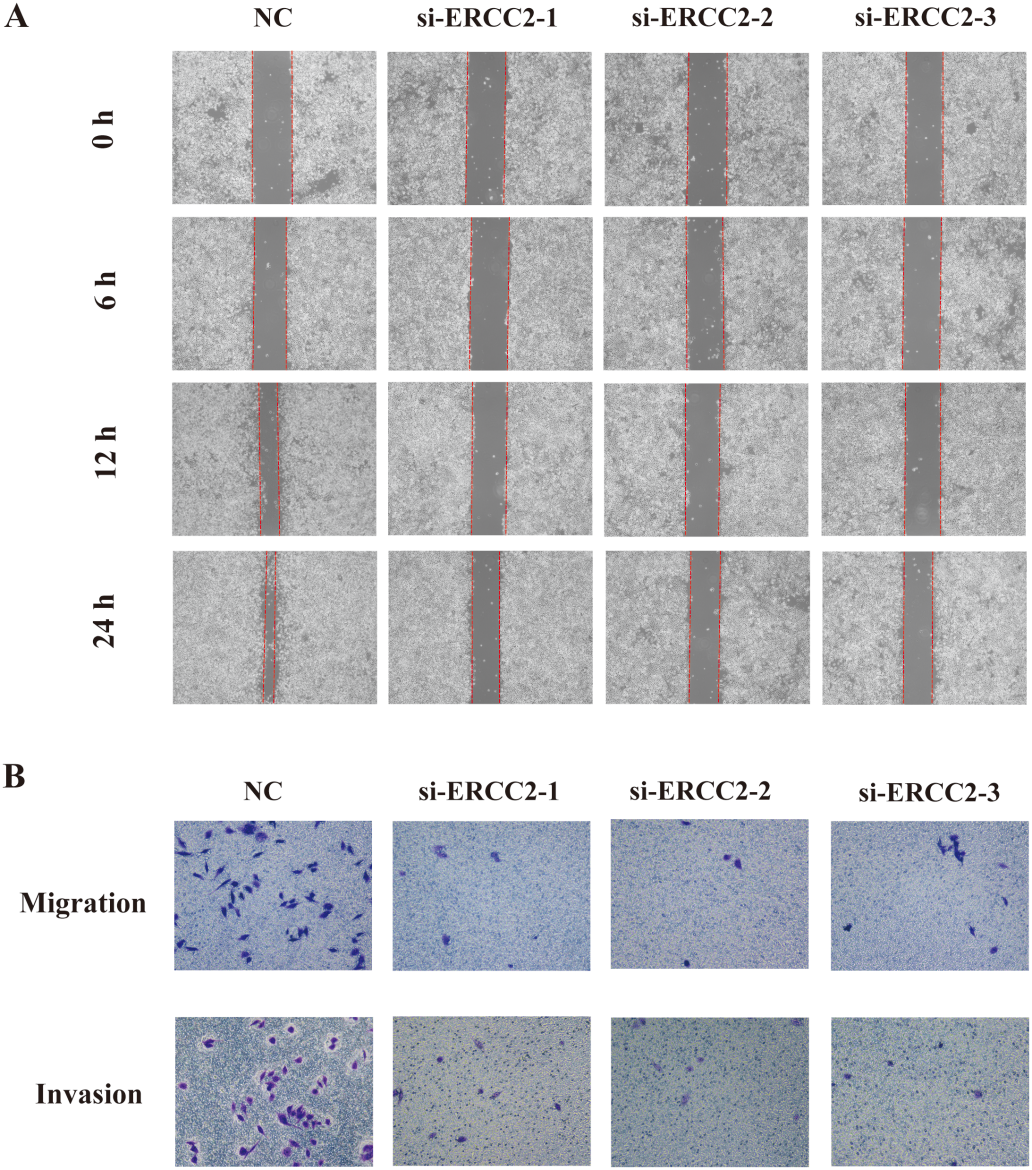
Knockdown of ERCC2 inhibits migration and invasion of bladder cancer cells. **(A)** Wound healing assay shows reduced wound closure in ERCC2 knockdown cells compared to control. **(B)** Transwell assay indicates decreased migration and invasion in ERCC2 knockdown cells compared to control.

To further explore the effects of ERCC2 on the migration and invasion of T24 cells, we performed wound healing assay and Transwell assay on T24 cells that were knocked down ERCC2. As shown in **Figure 15A**, the wounds in the control group healed significantly with the extension of time, while the experimental group did not show any significant wound healing, which indicated that the migratory ability of T24 cells after knocking down ERCC2 was significantly inhibited. Then the results of Transwell assay also confirmed the above conclusion that the migration and invasion abilities of T24 cells were significantly attenuated after knocking down ERCC2 (**Figure 15B**).The above results indicated that ERCC2 could effectively inhibit the migration and invasion of bladder cancer T24 cells.

## 4 Discussion

Our study underscores the importance of rigorous methodological approaches in bioinformatics research. By selecting patient samples with complete clinical and genomic data from the TCGA database and normalizing this data, we ensured the reliability and comparability of our gene expression analyses. Additionally, our use of the Benjamini-Hochberg procedure to adjust for multiple comparisons and the application of Cox proportional hazards models with multivariate adjustments for potential confounders provide a robust framework for assessing the prognostic significance of ERCC genes (23). Over the past decade, the genes ERCC1, ERCC2, and ERCC5 have garnered significant attention due to their crucial role in DNA repair processes. An increasing corpus of empirical research has provided substantial evidence supporting a robust association between the polymorphisms at the aforementioned genetic loci, which were previously hypothesized to influence protein functionality, and diverse forms of cancer. These include cancers affecting the larynx (24), prostate (25), skin, lung, and other diseases associated with smoking (26). The reduced efficacy of DNA repair is widely acknowledged to have a significant impact on the progression of cancer, as these deficits can accelerate genetic instability and increase the occurrence of genetic changes. Several studies have provided evidence supporting a favorable association between ERCC polymorphisms and elevated amounts of DNA adducts, which in turn are linked to an increased risk of cancer (27, 28). Multiple SNPs have been discovered within the ERCC group and extensively studied in many types of cancer, such as bladder cancer (29), laryngeal cancer, and extrahepatic bile duct cancer (30, 31). Despite the presence of previous evidence suggesting variations in ERCC expression in different types of cancer and its potential as a prognostic indicator in cancer therapy, a thorough and methodical examination of ERCC expression in pan-cancer and its prognostic importance has not been undertaken to date. We have systematically evaluated the expression patterns of ERCC family genes in cancer and integrated them in a multi-omics analysis.

A comprehensive evaluation was conducted to analyze the expression patterns of ERCC genes in cancer. Our findings indicate that ERCC1, 2, 3, 4, 5, 6, and 8 exhibited significantly elevated expression levels in numerous cancerous tissues when compared to their corresponding normal tissues. Notably, ERCC1 showed higher expression in cancer tissues than in normal tissues in all cancer types, whereas ERCC4 showed lower expression in cancer tissues than in normal tissues only in KIRC, KIRP, PRAD, THCA, and UCEC. ERCC1 is a valuable biomarker for platinum-based chemotherapy in cancer (32). The results indicate that a significant proportion of ERCC genes display higher expressions in various cancer tissues compared to their corresponding normal tissues. Subsequently, we found that of the seven ERCC family genes, ERCC1, 2, 3, 5, 6, and 8 showed differential expression in bladder cancer samples. Based on this initial premise, a comprehensive and meticulous investigation was undertaken to examine the patterns of ERCC expression in pan-cancer.

The ERCC is generally acknowledged for its significant contribution to the nucleotide excision repair (NER) process and the remediation of DNA damage. Several previous researches have displayed that ERCC genes may affect the pathogenesis of a variety of cancers and that there is an important association with survival and prognosis of cancer patients (33, 34). For example, previous researches have displayed significant correlations between single nucleotide polymorphisms in ERCC2, ERCC3, and ERCC4 and the risk of developing lung, skin, and breast cancers (35). It has also been reported in the literature that mutations in the ERCC2 gene increase the sensitivity to cisplatin in chemotherapy in patients with bladder cancer, that mutations in the ERCC3 gene increase the risk of breast and ovarian cancer, that ERCC4 correlates with overall survival in patients with ovarian cancer and in patients with gastric cancer, and that ERCC5 has been determined to be a prognostic indicator of survival in patients with ovarian cancer (33, 34, 36–39).

We analyzed the correlation between ERCC family genes and clinicopathological features or prognosis of pan-cancer. Through the utilization of KM plotter and univariate Cox regression analysis, we identified the potential prognostic significance of ERCC in pan-cancer. In addition, our research showed that some ERCC genes were useful for predicting OS, DFS, DSS, and PFS on their own. For example, in ACC, KIRC, LAML, and LUAD, higher ERCC1 expression was correlated with poorer overall survival (OS), suggesting that ERCC1 plays a tumor-promoting role in these cancers, whereas in BRCA and GBM, the expression level of ERCC1 was positively correlated with the patients’ overall survival (OS), indicating that ERCC1 plays a role in inhibiting tumor development. Similar findings were derived from our analysis of the data pertaining to PFS, DSS, and DFS. These results indicate that the expression of ERCC family genes holds promise as a prognostic marker for many types of cancer. To clarify, ERCCs have the potential to serve as indicators for predicting the clinicopathologic characteristics and survival prognosis of individuals diagnosed with cancer, albeit to a limited extent.

In our work, we analyzed the data of ERCCs in terms of mutations using public data obtained from open databases. The advancement of cancer is impacted by the cellular acquisition of genetic modifications, epigenetic alterations, transcriptome changes, and proteomic irregularities (40). Certain areas of the genome may exhibit either a loss or gain of function in somatic cells, thereby potentially indicating their involvement in either the suppression or promotion of cancer, respectively. Irreparable structural mutations in cells represent a significant underlying factor in the development of human cancers (41). Our findings suggest that amplification is the predominant form of gene mutation in ERCC families. The investigation of pan-cancer survival revealed a notable disparity in overall survival between patients with modified ERCC family genes and those with unmodified ERCCs, with the former exhibiting significantly lower survival rates. Consequently, ERCCs possess considerable promise as both biomarkers and targets for therapeutic interventions. However, more work is needed in the future to clarify how genetic polymorphisms affect tumorigenesis and progression.

The significant influence of the cellular constituents inside the tumor microenvironment (TME) on both treatment resistance and the distinctive symptoms of cancer is widely acknowledged (42). While various genetic alterations are employed by cancer cells to launch the development of cancer, alterations in the microenvironment around the tumor may be prevalent across different types of tumors. This situation provides a potential avenue for therapeutic intervention that focuses on addressing these processes (43). Cancer stem cells have a capacity for self-renewal that distinguishes them from typical cancer cells. This property is known to have important implications in many ways (44). The tumor stem cell index represents a novel metric for assessing the advancement of tumors. Numerous studies have provided evidence of a negative correlation between the tumor stem cell index and survival rates, suggesting that elevated tumor stem cell indices are linked to the presence of metastatic malignancies (45). In the context of head and neck squamous cell carcinoma, it has been observed that there is a significant correlation between an elevated tumor stem cell index and the presence of mutations in the NSD1 gene (46). Our study revealed a favorable correlation between the expression of ERCC2 and ERCC6 in patients with bladder cancer (BLCA) and the BLCA stem cell index. This finding suggests that ERCC2 and ERCC6 can be used to some extent as prognostic survival indicators in BLCA patients. Furthermore, our research reveals a negative association between the levels of expression of most ERCC family genes with both stromal score and immunological score. This finding indicates that an elevated expression of these bigger ERCC genes is correlated with a higher abundance of immune and stromal cells, as well as a reduced abundance of tumor cells.

While our study provides valuable insights into the role of ERCC genes in cancer, we acknowledge several limitations that may impact the interpretation and generalizability of our findings. Our study relies heavily on data obtained from public databases such as TCGA and UCSC Xena. These datasets are invaluable resources but are not without limitations. Selection bias and inconsistencies in data collection and processing across different studies may introduce variability. For instance, differences in sample handling, sequencing techniques, and data annotation can affect the comparability of datasets. Future studies should aim to validate our findings using independent cohorts and more controlled data collection protocols. The inclusion of a wide range of cancer types in our pan-cancer analysis introduces another layer of complexity. Cancer is a highly heterogeneous disease, and the biological behavior of tumors can vary significantly not only between different cancer types but also within subtypes of the same cancer. This heterogeneity can obscure specific associations and biological mechanisms pertinent to individual cancer types. Therefore, while pan-cancer analyses provide broad insights, detailed studies focusing on specific cancer types are necessary to understand the unique roles of ERCC genes in those contexts. The variability in treatment protocols across different cancer types and institutions is another critical factor. Our study did not account for differences in treatment regimens, which can influence the expression and mutation profiles of ERCC genes. These variations could potentially affect the prognostic and therapeutic relevance of ERCC gene alterations. Future research should incorporate treatment-related variables to better understand the impact of therapy on ERCC gene function and patient outcomes.

In this study, we examined the expression and prognostic significance of ERCC genes across various tumor types, emphasizing the differences among the ERCC genes and the cancers in which they appear. While numerous studies have focused on individual ERCC genes in specific cancers or all ERCC genes within one cancer type, our research aims to explore the comprehensive impact of ERCC gene expression variations on clinical outcomes across a broad spectrum of cancers. We present detailed analyses of pan-cancer patterns as well as those specific to individual tumor types. Our findings offer a foundation for further research into the role of ERCC genes in cancer progression and prognosis. From an integrated perspective utilizing multiple omics and datasets, we observed that ERCC-family genes are significantly associated with clinicopathological factors, treatment responses, and tumor immunological characteristics. Additionally, our *in vitro* assays showed that knockdown of ERCC2 expression inhibited the proliferation, migration, and invasion of bladder cancer cells. There is reason to believe that ERCC2 may be a potential therapeutic target for bladder cancer. This study systematically analyzes the potential of ERCC family members as diagnostic and prognostic biomarkers and therapeutic targets using a multi-omics approach, identifying new targets for future drug therapies.

## 5 Conclusion

This study provides novel insights into the role of ERCC genes in cancer progression and highlights their potential as targets for personalized cancer therapy. Our findings suggest that ERCC genes could serve as valuable prognostic biomarkers and therapeutic targets. Further research is warranted to fully elucidate the mechanisms underlying ERCC gene functions in cancer progression and treatment.

## Data Availability

The datasets presented in this study can be found in online repositories. The names of the repository/repositories and accession number(s) can be found in the article/**Supplementary Material**.

## Supplementary material

Supplementary Figure 1 Differential expression of ERCC in pan-cancer. Box plots illustrating the expression levels of ERCC genes in various cancer types.

Supplementary Figure 2 ERCC gene expression in cancer and normal tissues. Box plots showing the differential expression of ERCC genes in cancerous versus normal tissues.

Supplementary Figure 3 Differential expression of ERCC at different stages. Analysis of ERCC gene expression across various stages of cancer.

Supplementary Figure 4 ERCC gene expression and treatment response. Association between ERCC gene expression levels and response to initial treatment.

Supplementary Figure 5 Mutation frequency of ERCC in pan-cancer. Analysis of the frequency of mutations in ERCC genes across different cancer types.

Supplementary Figure 6 Comparison of mutated and unmutated groups on OS. Kaplan-Meier survival curves comparing overall survival between patients with and without ERCC gene mutations.

Supplementary Figure 7 Associations between ERCC mRNA expression and single nucleotide variants (SNVs). Analysis of the correlation between ERCC mRNA expression and SNV frequency.

Supplementary Figure 8 Relationship between ERCC genes and common immune checkpoints. Heatmap illustrating the associations between ERCC gene expression and 47 common immune checkpoints in various cancers.

Supplementary Figure 9 Association analysis between ERCC expression and tumor mutational burden (TMB). Satellite maps showing the correlation between ERCC gene expression and TMB across different cancer types.

Supplementary Figure 10 Association analysis between ERCC expression and microsatellite instability (MSI). Satellite maps illustrating the relationship between ERCC gene expression and MSI in various cancers.

Supplementary Figure 11 GSEA enrichment analyses of ERCC family genes expression based on the KEGG pathways in bladder cancer. GSEA results showing the enrichment of KEGG pathways in bladder cancer for ERCC gene expression.

Supplementary Figure 12 GSEA enrichment analyses of ERCC family genes expression based on the Hallmark pathways in bladder cancer. GSEA results illustrating the enrichment of Hallmark pathways in bladder cancer for ERCC gene expression.

Supplementary Figure 13 Correlation of ERCC gene expression with clinicopathologic features and immune subtypes in bladder cancer. **(A)** Differential expression of ERCC genes across different immune subtypes. **(B)** Comparison of ERCC gene expression in high-grade versus low-grade bladder cancer. **(C)** Comparison of ERCC gene expression in papillary versus non-papillary bladder cancer.

Supplementary Table 1 K-M analysis of OS, PFS, DFS, and DSS for 33 cancer types summarized by ERCC expression.

Supplementary Table 2 Drug sensitivity analysis of ERCC family genes based on the CellMiner database.

Supplementary Table 3 The GSEA was performed for each ERCC gene using the KEGG pathways based on 33 different cancer datasets.

Supplementary Table 4 The GSEA was performed for each ERCC gene using the HallMarker gene sets acorss 33 different cancer datasets.
