# Supplementary figures and images for: Bioinformatics analysis of ERCC family in pan-cancer and ERCC2 in bladder cancer
Source: Front Immunol. 2024 Aug 13;15:1402548. doi: 10.3389/fimmu.2024.1402548 (PMC11347307; doi:10.3389/fimmu.2024.1402548)

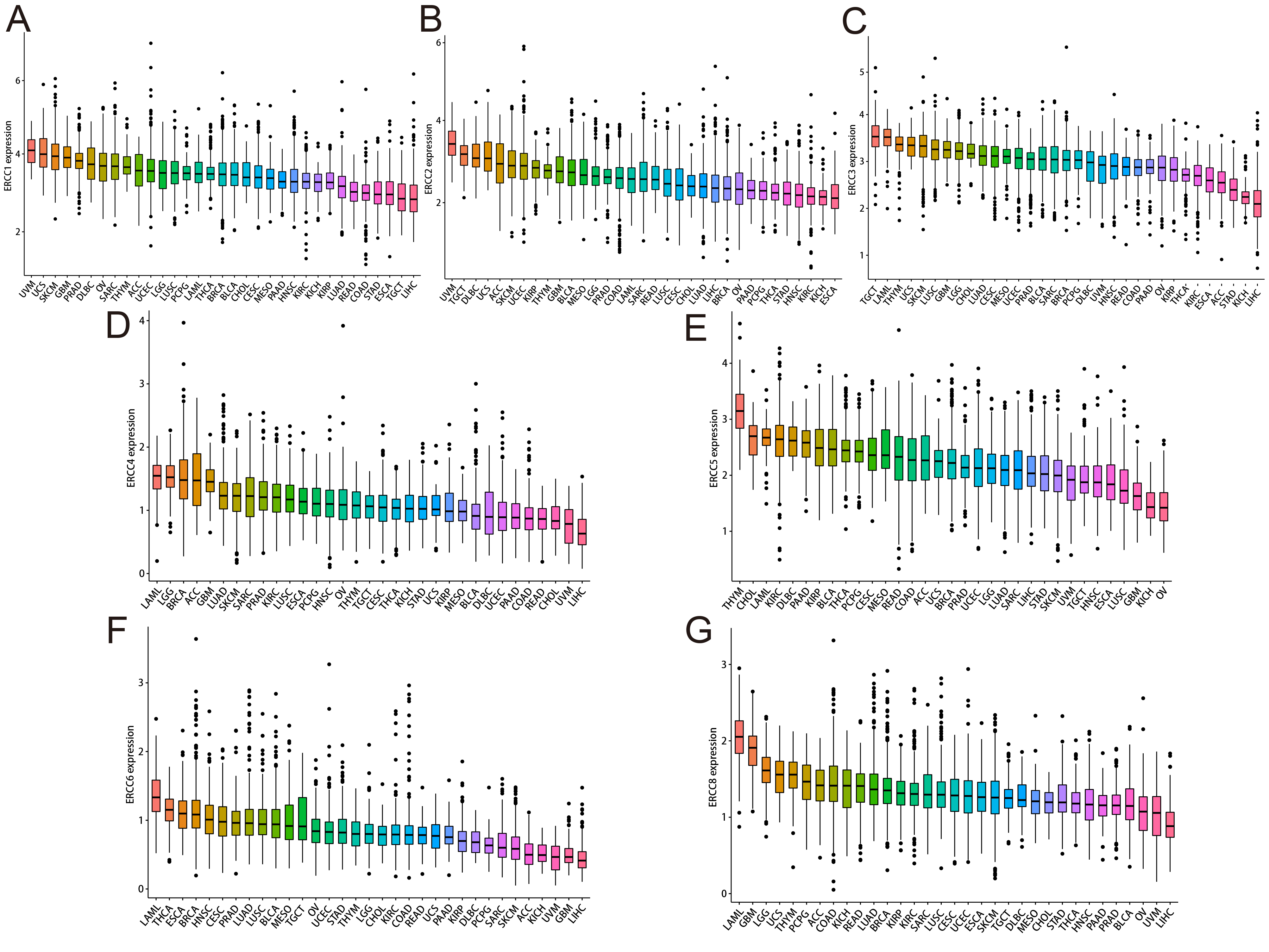

Supplement: Supplementary Figure 1 — Differential expression of ERCC in pan-cancer. Box plots illustrating the expression levels of ERCC genes in various cancer types. [file Image1.tif]

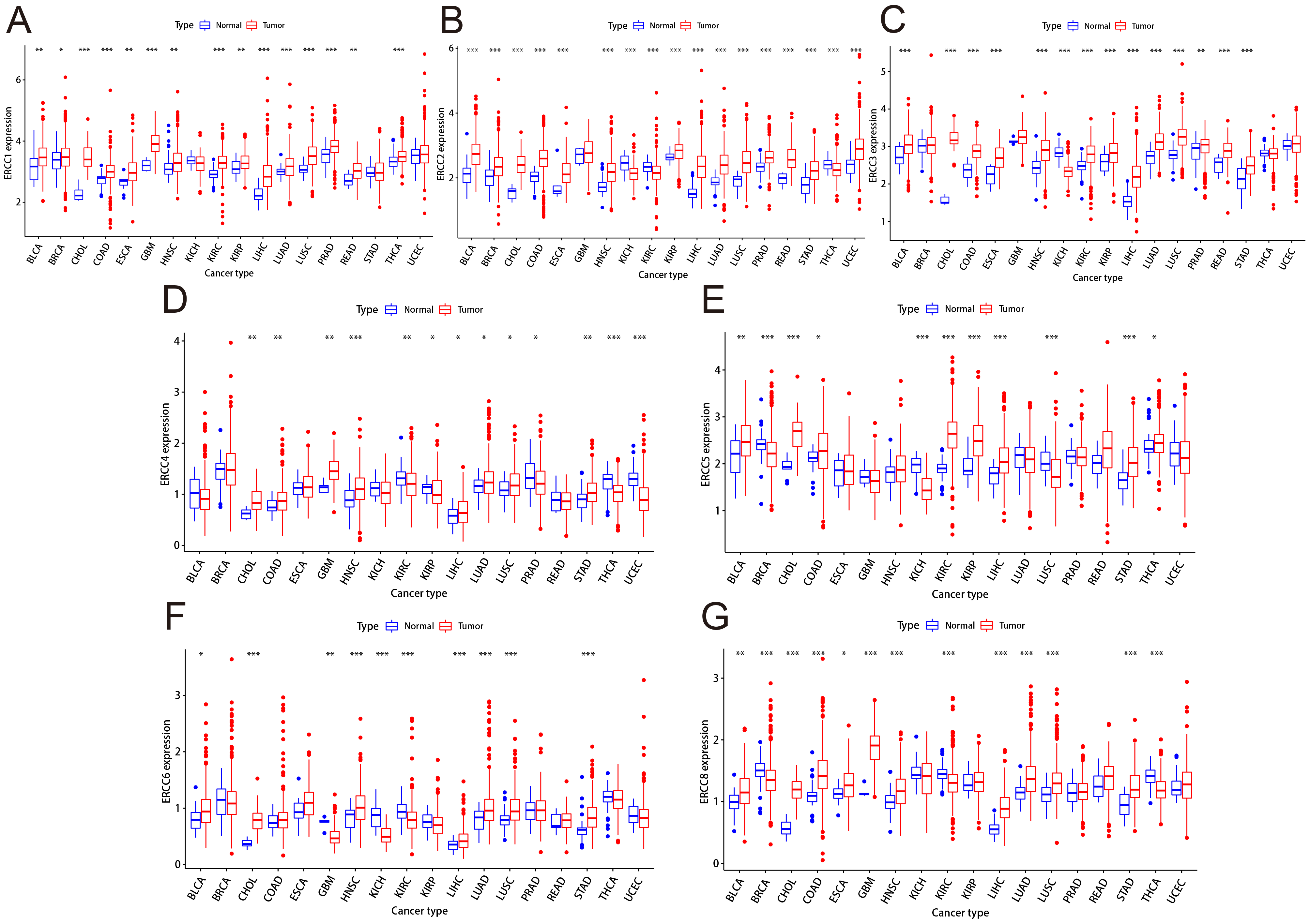

Supplement: Supplementary Figure 2 — ERCC gene expression in cancer and normal tissues. Box plots showing the differential expression of ERCC genes in cancerous versus normal tissues. [file Image2.tif]

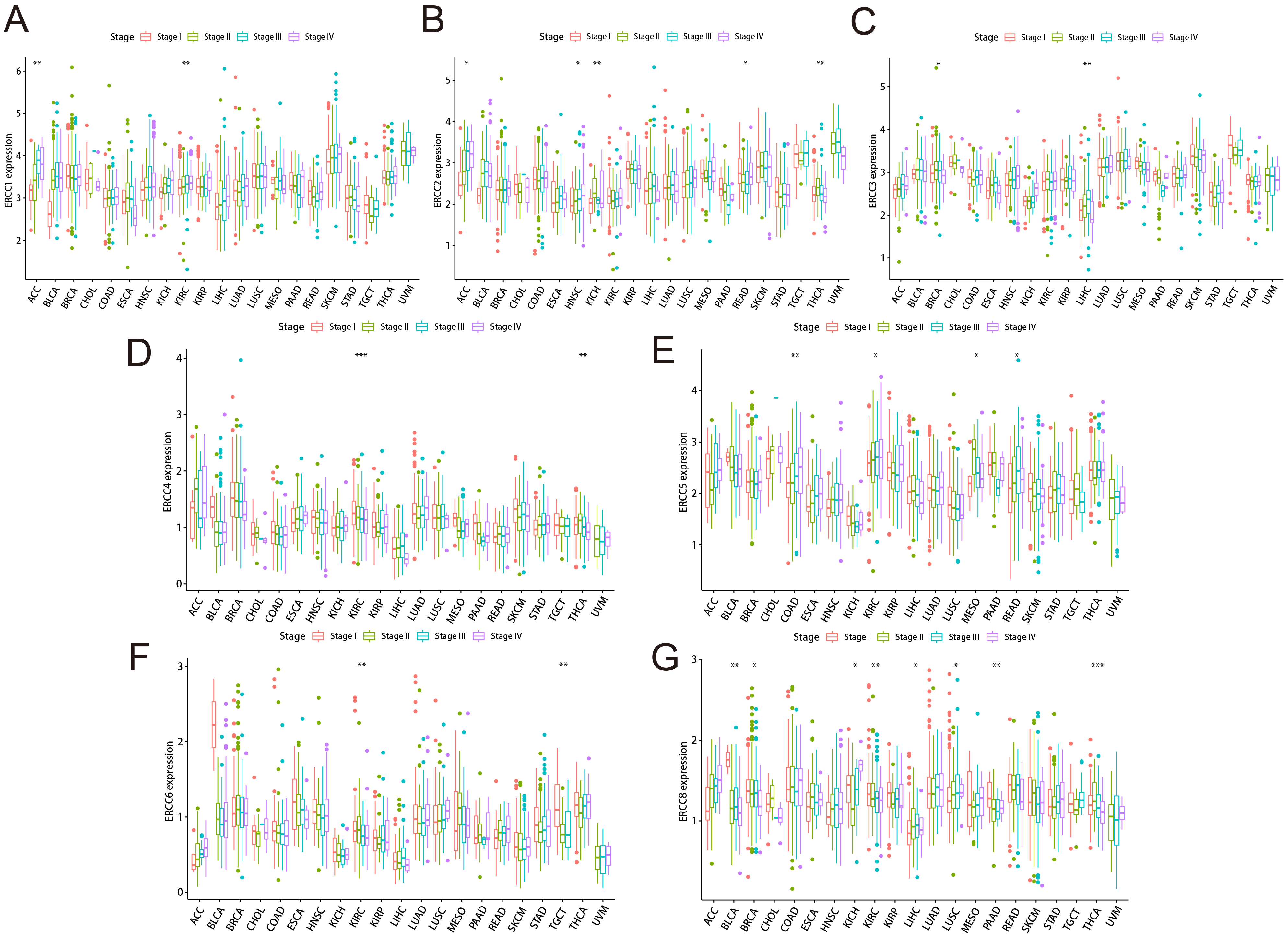

Supplement: Supplementary Figure 3 — Differential expression of ERCC at different stages. Analysis of ERCC gene expression across various stages of cancer. [file Image3.tif]

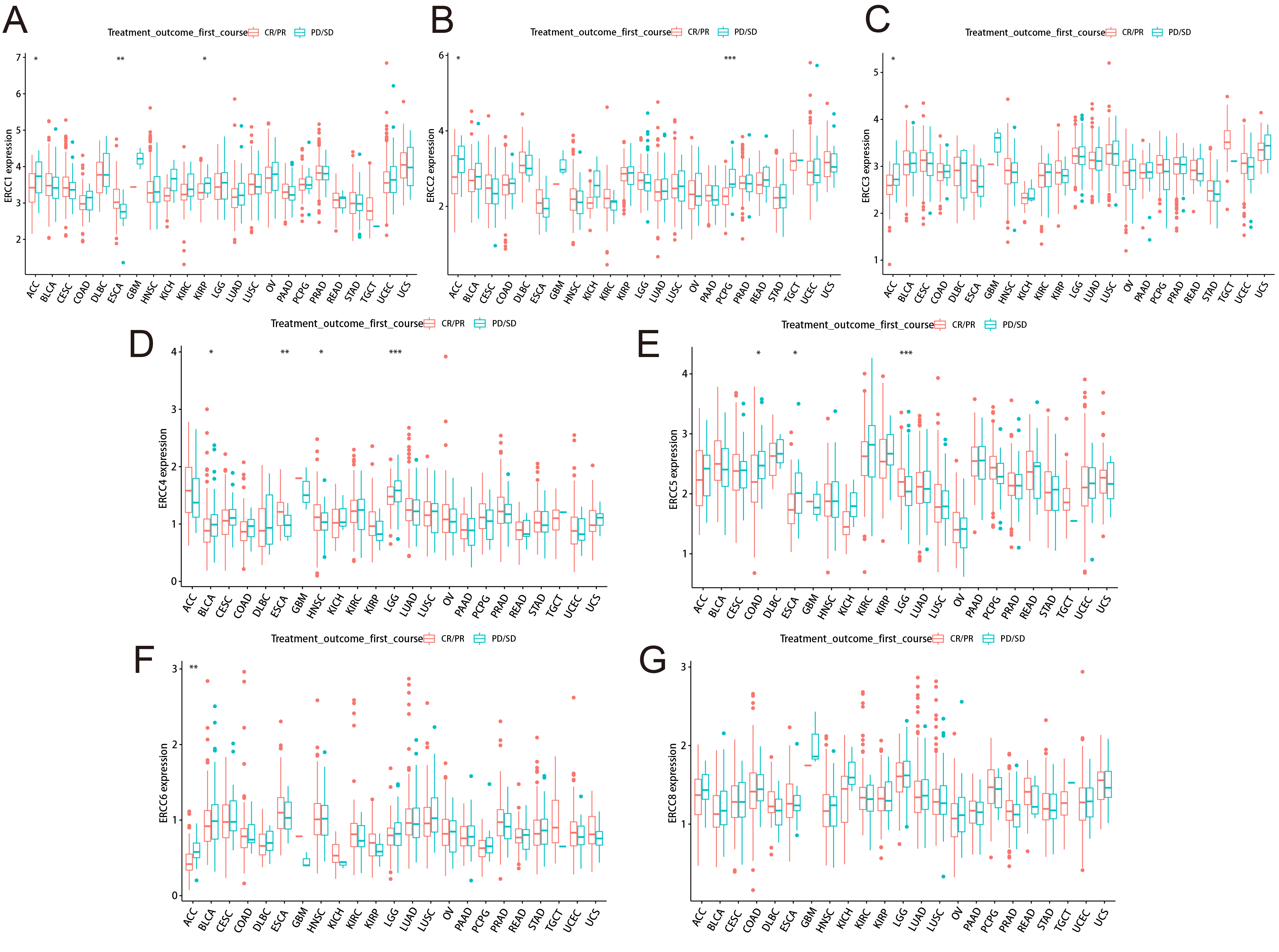

Supplement: Supplementary Figure 4 — ERCC gene expression and treatment response. Association between ERCC gene expression levels and response to initial treatment. [file Image4.tif]

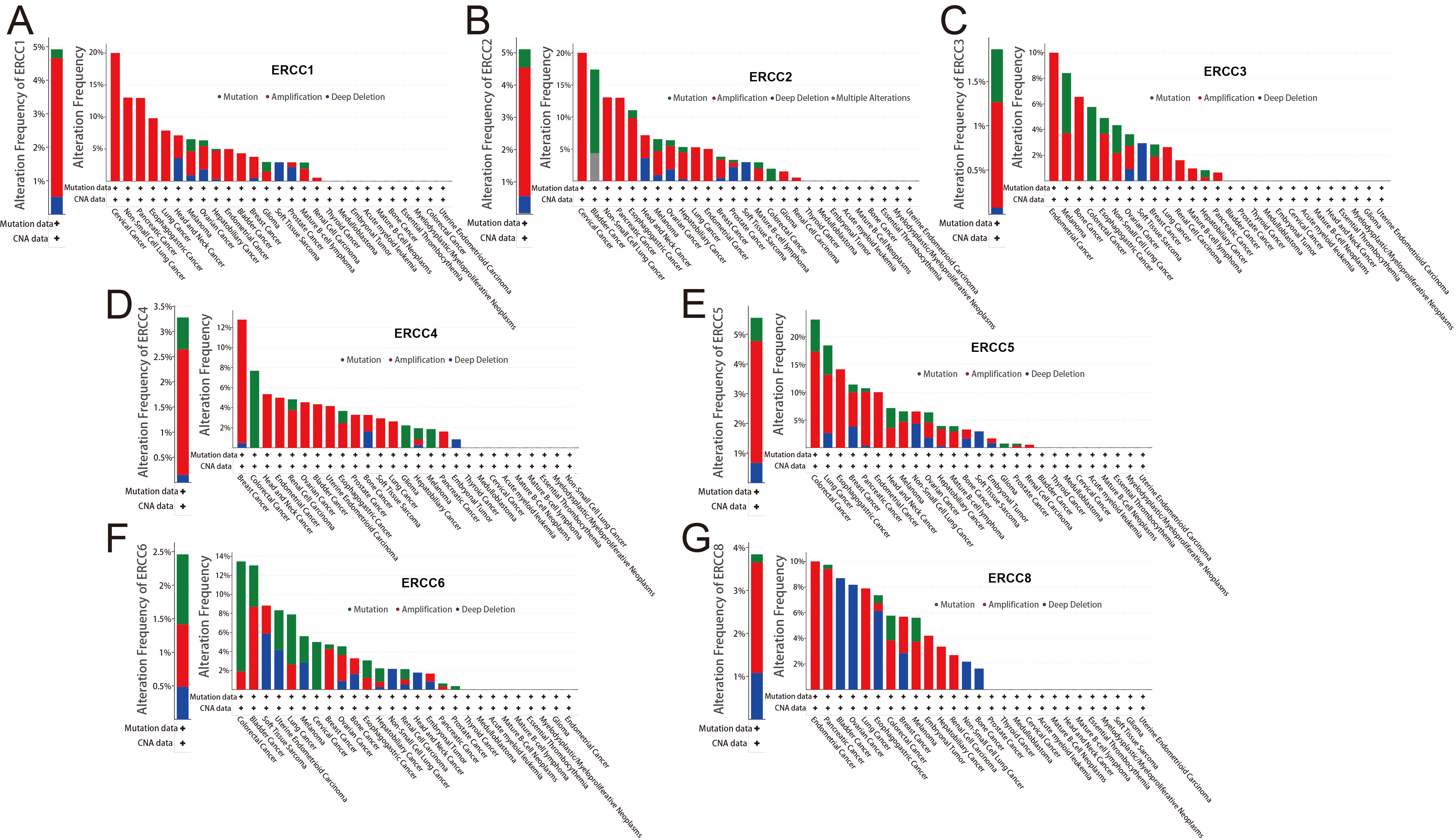

Supplement: Supplementary Figure 5 — Mutation frequency of ERCC in pan-cancer. Analysis of the frequency of mutations in ERCC genes across different cancer types. [file Image5.tif]

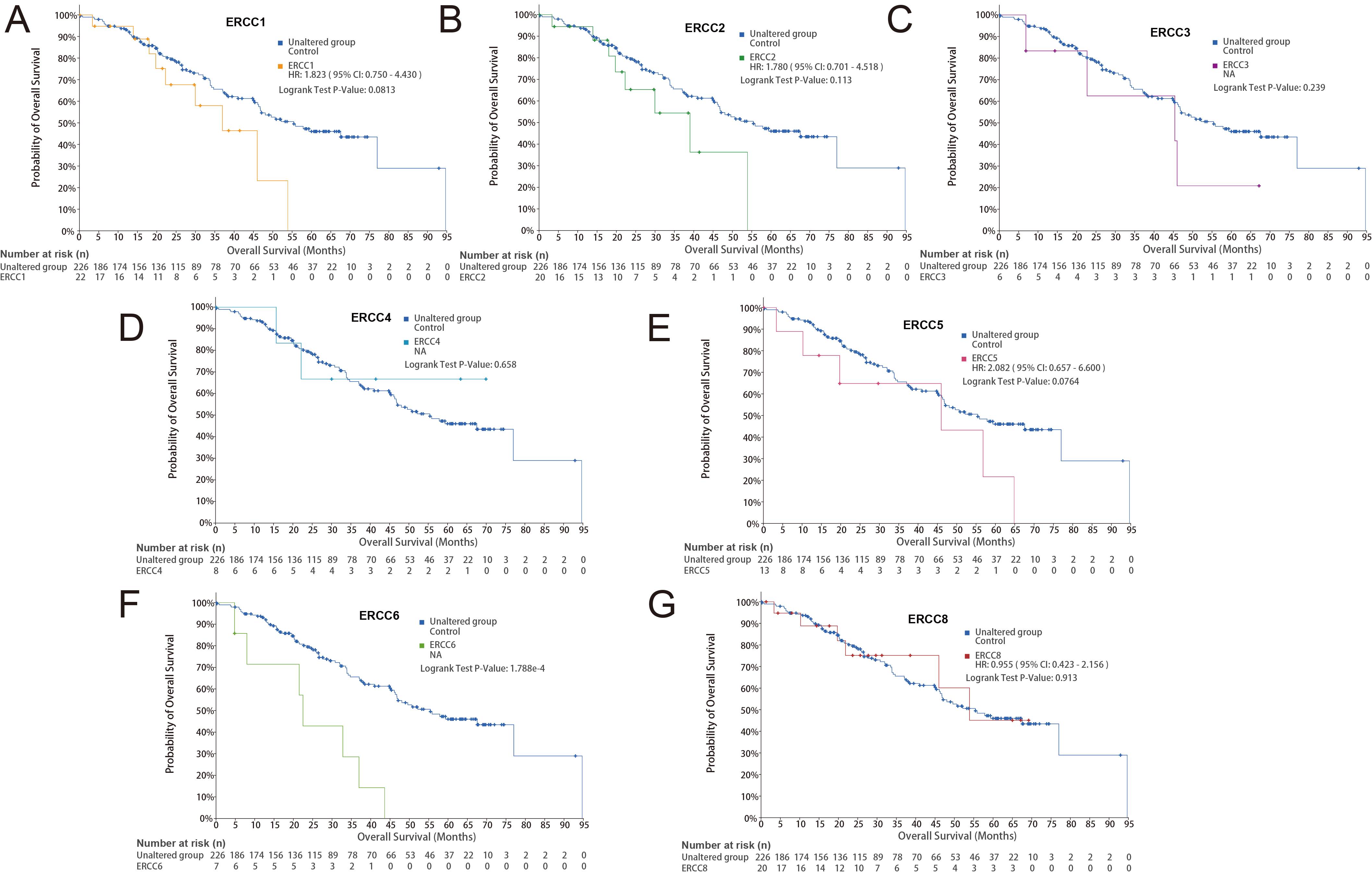

Supplement: Supplementary Figure 6 — Comparison of mutated and unmutated groups on OS. Kaplan-Meier survival curves comparing overall survival between patients with and without ERCC gene mutations. [file Image6.tif]

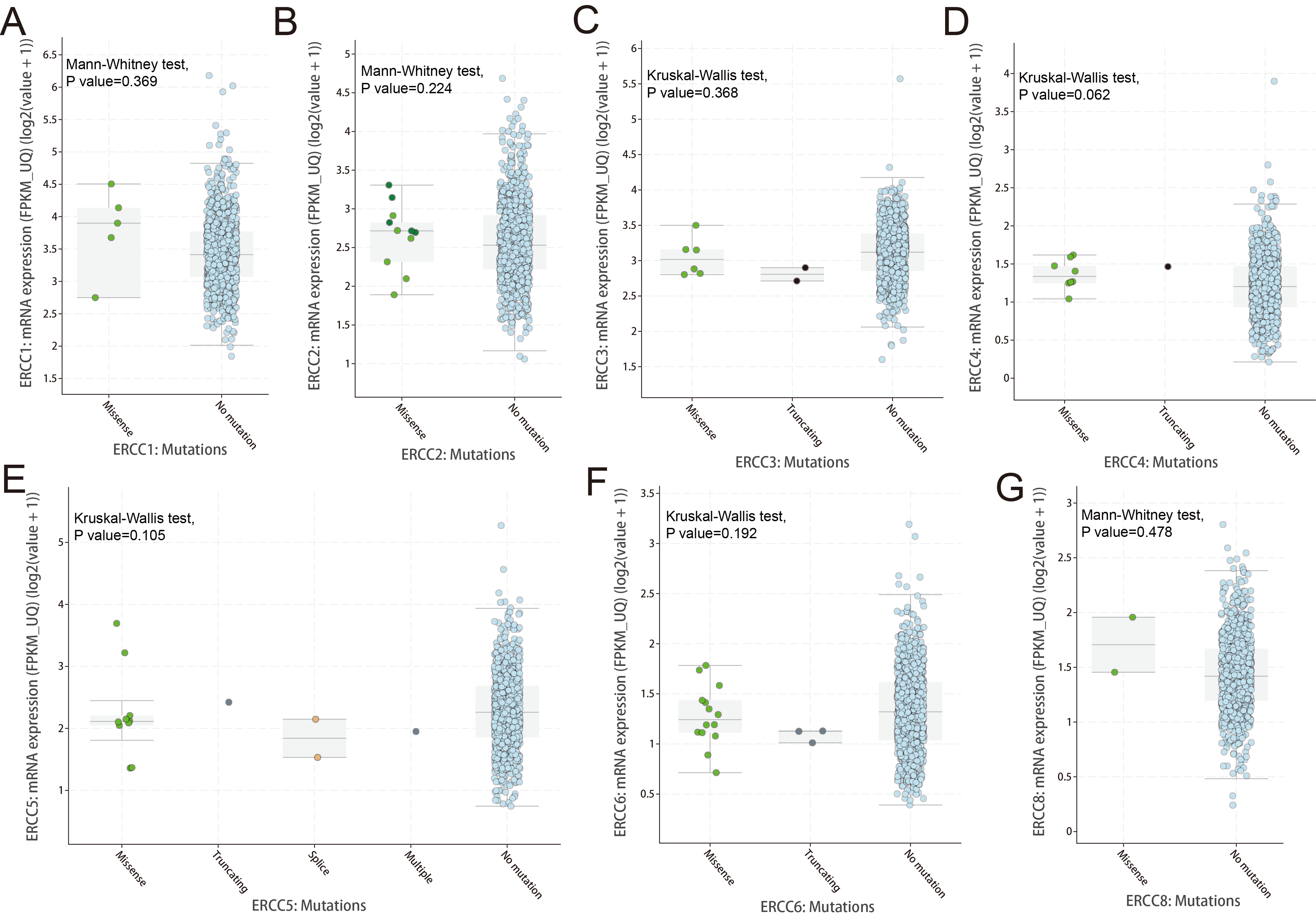

Supplement: Supplementary Figure 7 — Associations between ERCC mRNA expression and single nucleotide variants (SNVs). Analysis of the correlation between ERCC mRNA expression and SNV frequency. [file Image7.tif]

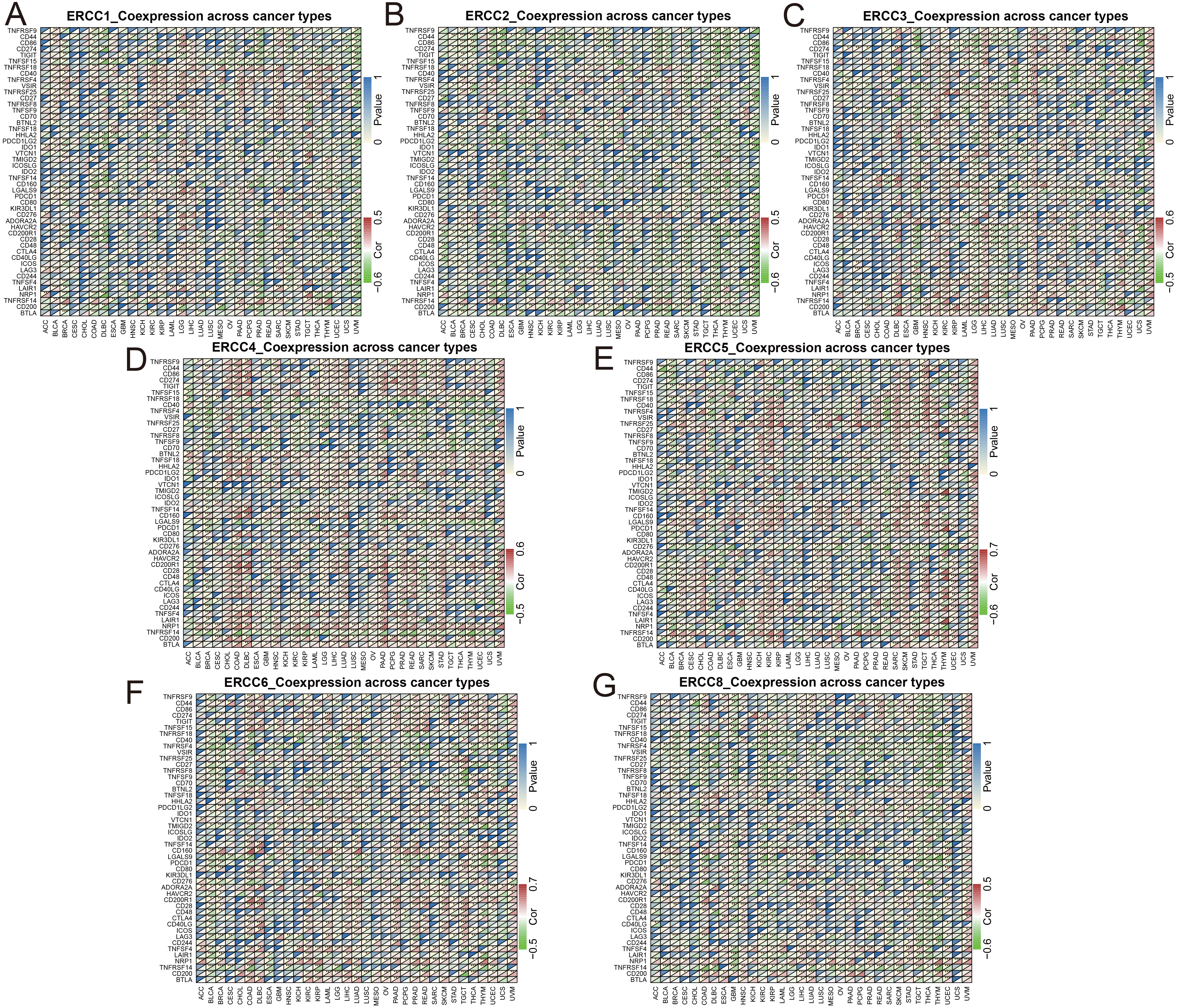

Supplement: Supplementary Figure 8 — Relationship between ERCC genes and common immune checkpoints. Heatmap illustrating the associations between ERCC gene expression and 47 common immune checkpoints in various cancers. [file Image8.tif]

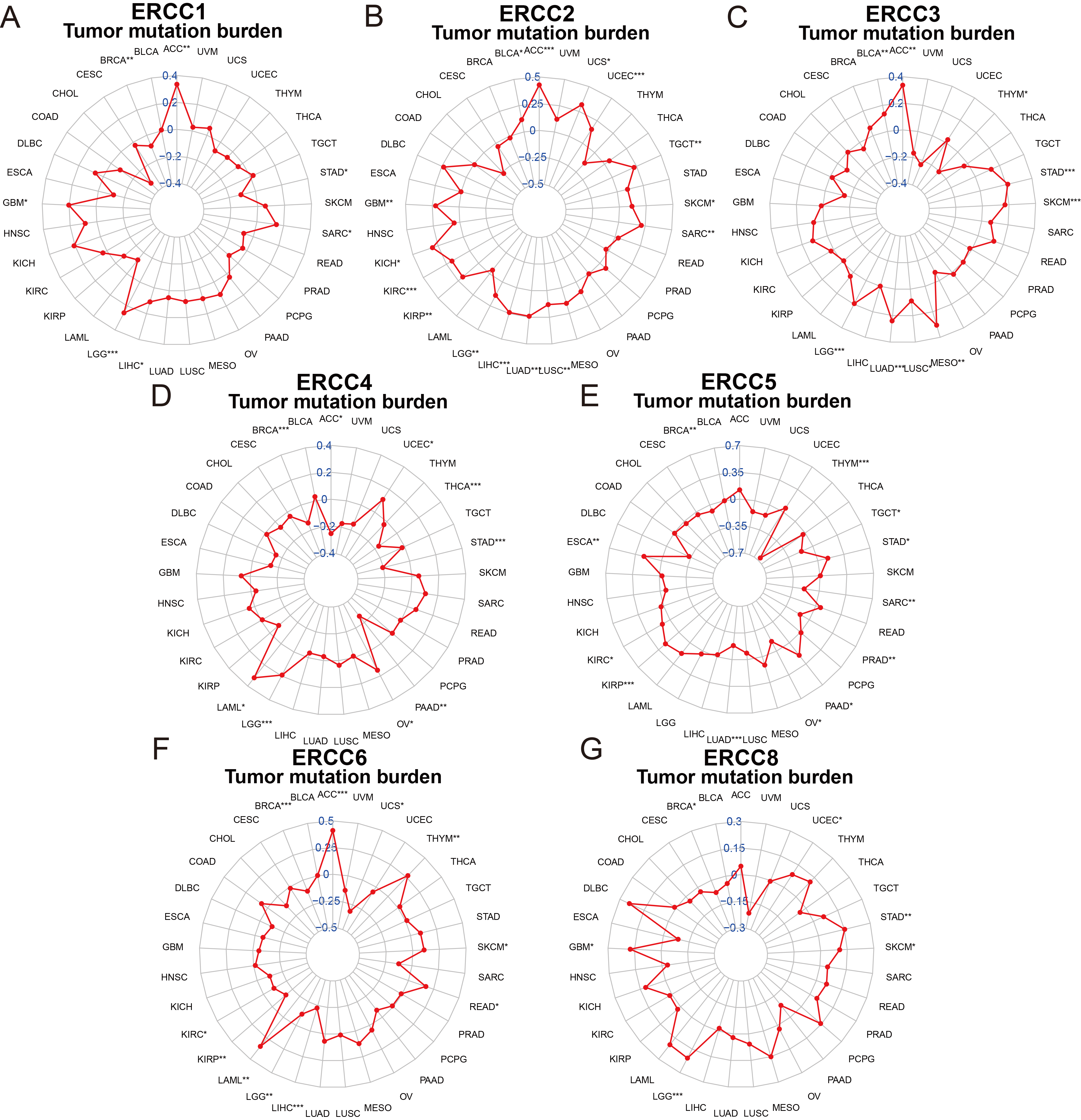

Supplement: Supplementary Figure 9 — Association analysis between ERCC expression and tumor mutational burden (TMB). Satellite maps showing the correlation between ERCC gene expression and TMB across different cancer types. [file Image9.tif]

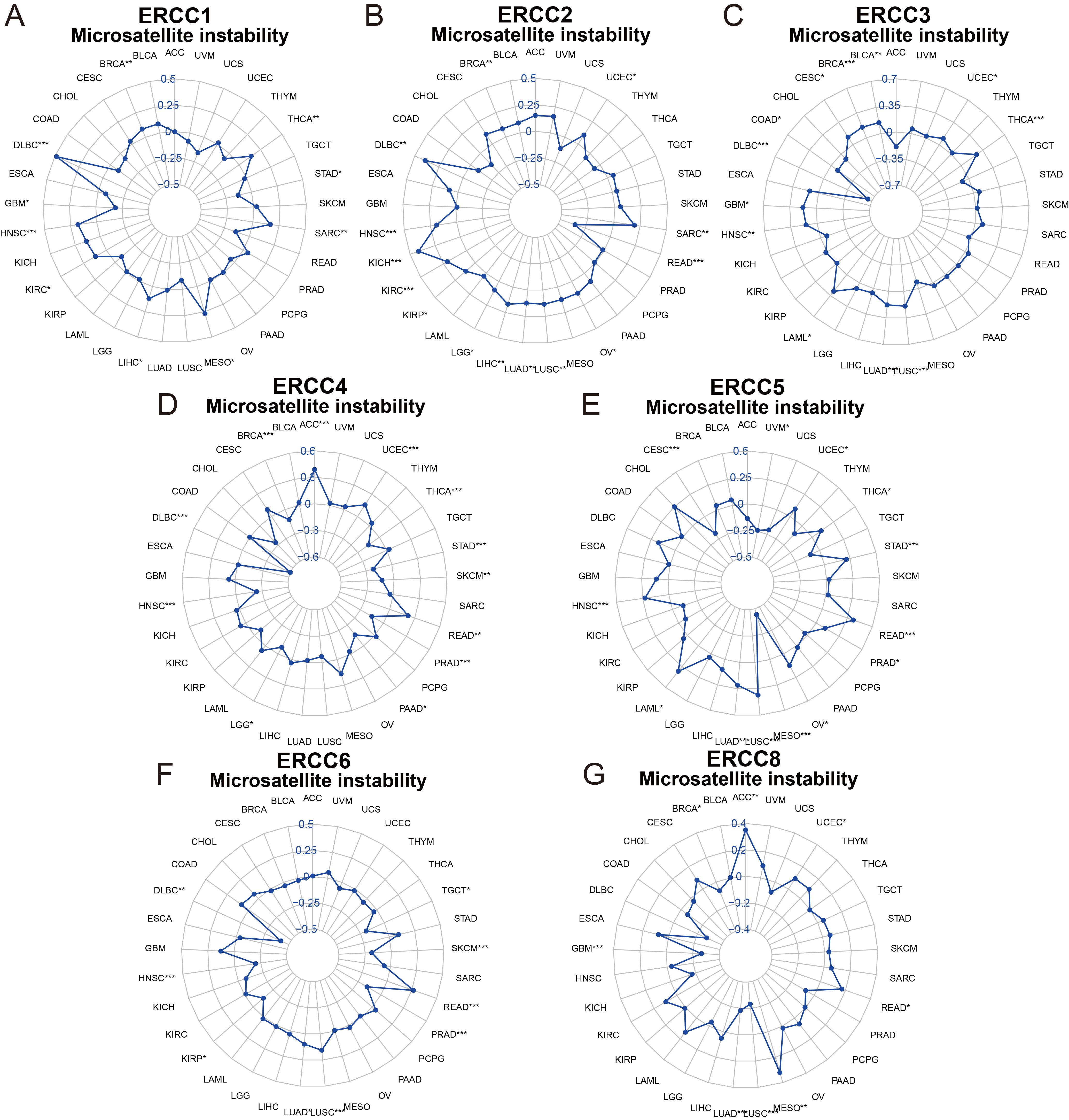

Supplement: Supplementary Figure 10 — Association analysis between ERCC expression and microsatellite instability (MSI). Satellite maps illustrating the relationship between ERCC gene expression and MSI in various cancers. [file Image10.tif]

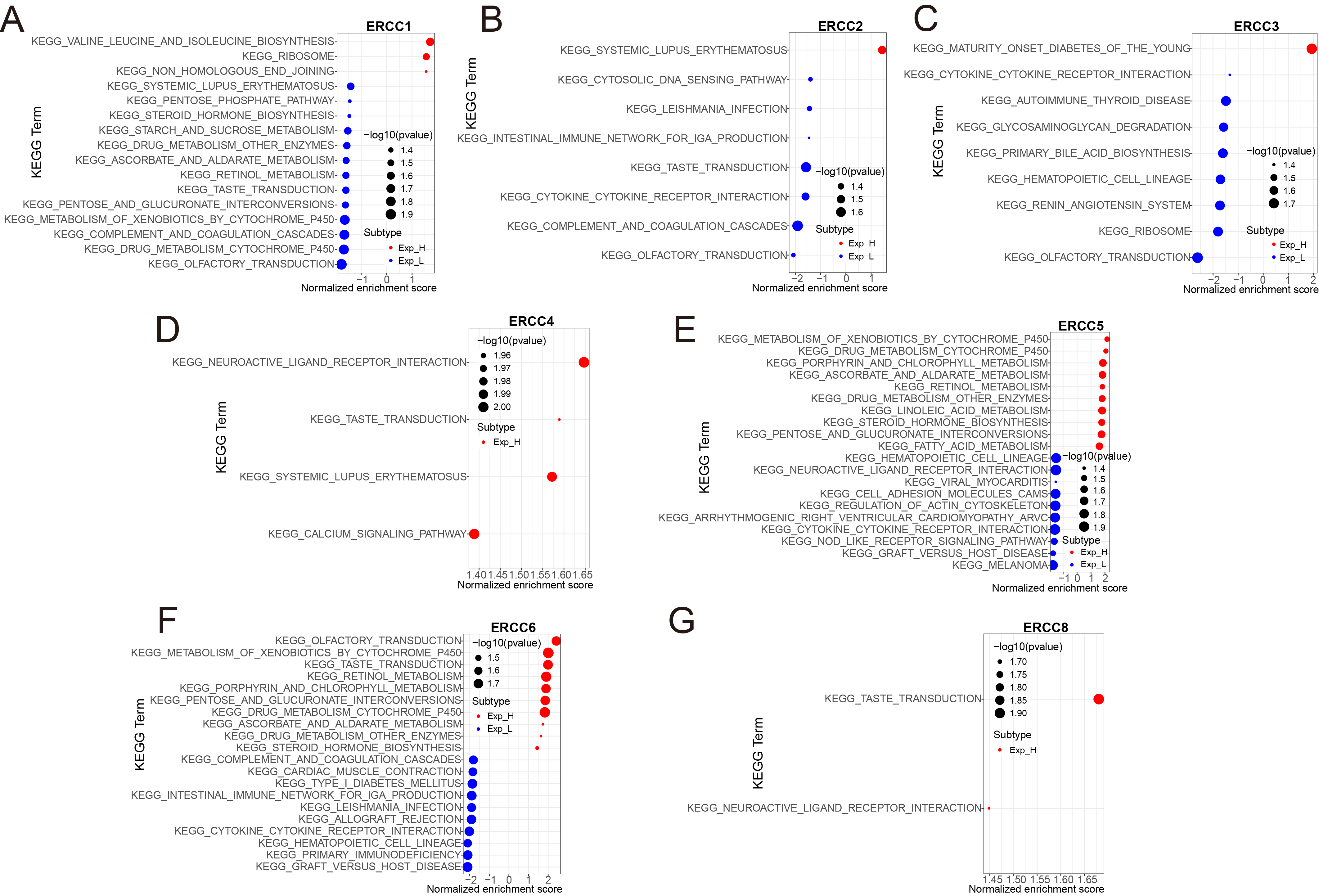

Supplement: Supplementary Figure 11 — GSEA enrichment analyses of ERCC family genes expression based on the KEGG pathways in bladder cancer. GSEA results showing the enrichment of KEGG pathways in bladder cancer for ERCC gene expression. [file Image11.tif]

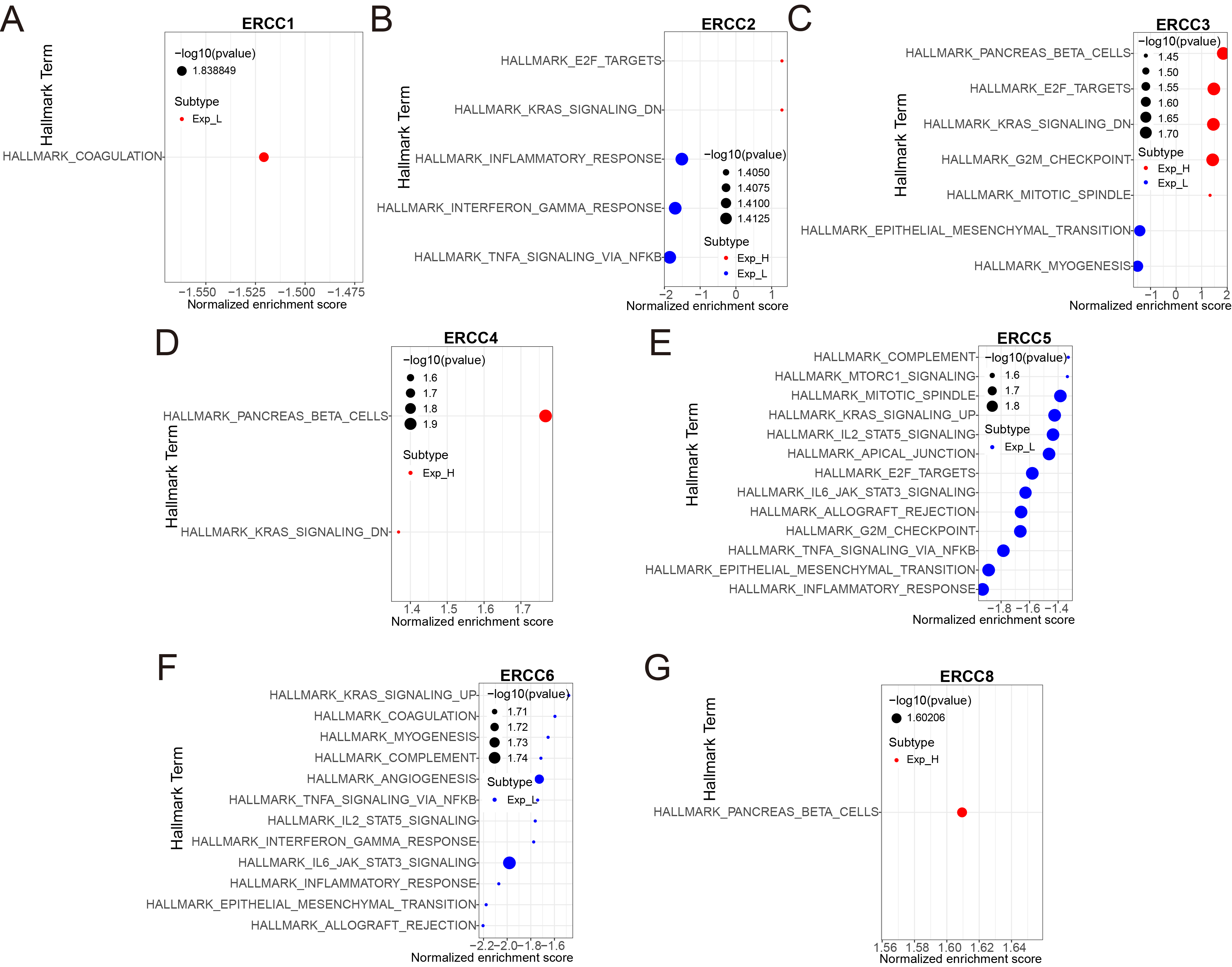

Supplement: Supplementary Figure 12 — GSEA enrichment analyses of ERCC family genes expression based on the Hallmark pathways in bladder cancer. GSEA results illustrating the enrichment of Hallmark pathways in bladder cancer for ERCC gene expression. [file Image12.tif]

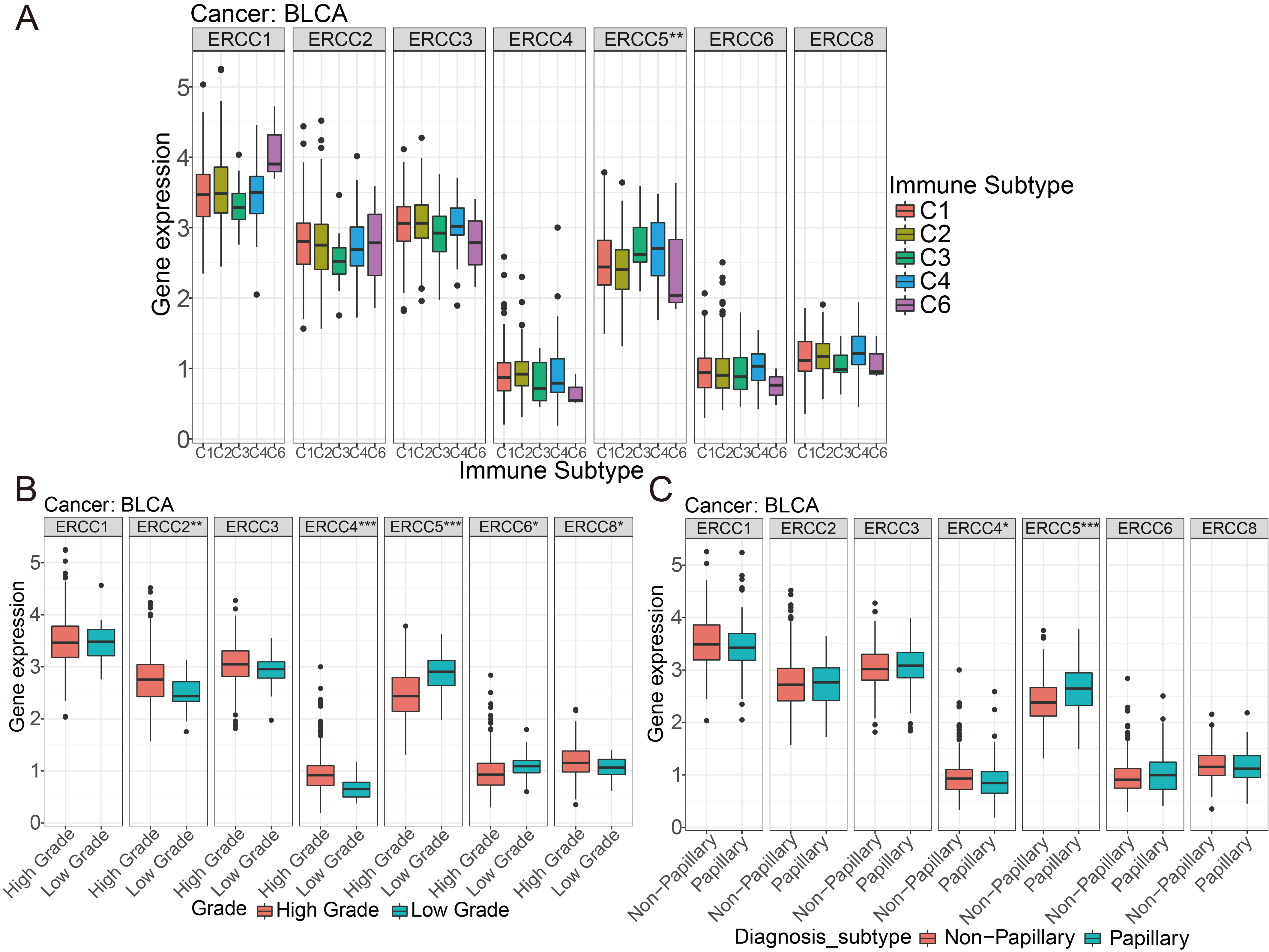

Supplement: Supplementary Figure 13 — Correlation of ERCC gene expression with clinicopathologic features and immune subtypes in bladder cancer. (A) Differential expression of ERCC genes across different immune subtypes. (B) Comparison of ERCC gene expression in high-grade versus low-grade bladder cancer. (C) Comparison of ERCC gene expression in papillary versus non-papillary bladder cancer. [file Image13.tif]
